# Can a serious game-based cognitive training attenuate cognitive decline related to Alzheimer’s disease? Protocol for a randomized controlled trial

**DOI:** 10.1186/s12888-022-04131-7

**Published:** 2022-08-12

**Authors:** Esther Brill, Christine Krebs, Michael Falkner, Jessica Peter, Katharina Henke, Marc Züst, Lora Minkova, Anna-Katharine Brem, Stefan Klöppel

**Affiliations:** 1 University Hospital of Old Age Psychiatry and Psychotherapy, University of Bern, Bern, Switzerland; 2 Swiss Institute for Translational and Entrepreneurial Medicine, Bern, Switzerland; 3 Graduate School for Health Sciences, University of Bern, Bern, Switzerland; 4 ARTORG Center for Biomedical Engineering Research, University of Bern, Bern, Switzerland; 5 Cognitive Neuroscience of Memory and Consciousness, Institute of Psychology, University of Bern, Bern, Switzerland; 6 Department of Old Age Psychiatry, Institute of Psychiatry, Psychology and Neuroscience, King’s College London, London, UK

**Keywords:** Computerized cognitive training, Alzheimer’s disease, Mild cognitive impairment, Subjective cognitive decline, Serious games, Cognitive training, Magnetic resonance imaging, Training adherence

## Abstract

**Background:**

Alzheimer’s disease (AD) is a major public health issue. Cognitive interventions such as computerized cognitive trainings (CCT) are effective in attenuating cognitive decline in AD. However, in those at risk of dementia related to AD, results are heterogeneous. Efficacy and feasibility of CCT needs to be explored in depth. Moreover, underlying mechanisms of CCT effects on the three cognitive domains typically affected by AD (episodic memory, semantic memory and spatial abilities) remain poorly understood.

**Methods:**

In this bi-centric, randomized controlled trial (RCT) with parallel groups, participants (planned *N* = 162, aged 60–85 years) at risk for AD and with at least subjective cognitive decline will be randomized to one of three groups. We will compare serious game-based CCT against a passive wait list control condition and an active control condition (watching documentaries). Training will consist of daily at-home sessions for 10 weeks (50 sessions) and weekly on-site group meetings. Subsequently, the CCT group will continue at-home training for an additional twenty-weeks including monthly on-site booster sessions. Investigators conducting the cognitive assessments will be blinded. Group leaders will be aware of participants’ group allocations. Primarily, we will evaluate change using a compound value derived from the comprehensive cognitive assessment for each of three cognitive domains. Secondary, longitudinal functional and structural magnetic resonance imaging (MRI) and evaluation of blood-based biomarkers will serve to investigate neuronal underpinnings of expected training benefits.

**Discussion:**

The present study will address several shortcomings of previous CCT studies. This entails a comparison of serious game-based CCT with both a passive and an active control condition while including social elements crucial for training success and adherence, the combination of at-home and on-site training, inclusion of booster sessions and assessment of physiological markers. Study outcomes will provide information on feasibility and efficacy of serious game-based CCT in older adults at risk for AD and will potentially generalize to treatment guidelines. Moreover, we set out to investigate physiological underpinnings of CCT induced neuronal changes to form the grounds for future individually tailored interventions and neuro-biologically informed trainings.

**Trial registration:**

This RCT was registered 1st of July 2020 at clinicaltrials.gov (Identifier NCT04452864).

**Supplementary Information:**

The online version contains supplementary material available at 10.1186/s12888-022-04131-7.

## Introduction

### Background

Alzheimer’s disease (AD) is a leading cause of disability and a major health- and societal issue. Although the clinical presentation at mild or early stages may vary [1], AD typically affects three cognitive domains: episodic memory (EM), semantic memory (SM), and visuospatial abilities (SA [2];). The pathophysiological chain of events in neurodegenerative dementias commences decades before the onset of clinically relevant symptoms [3]. Of note is that subjective cognitive decline (SCD), defined as self-perceived decrease in cognitive performance, is considered an early at-risk state for progression to AD especially when the perceived decline is worrisome [4]. Here, despite scoring within normal range in neuropsychological test, people with SCD have a somewhat altered cognitive performance profile with lower results in EM tasks compared to healthy controls [5, 6].

The long phase before onset opens the possibility to alter the disease course and to postpone symptom manifestation. To date, pharmacological agents to counteract early cognitive dysfunction in the elderly are limited [7, 8], which gave rise to the development of cognitive interventions. As one sub-category of cognitive interventions, cognitive stimulation is defined by the activation of broad memory patterns and is well established in clinical settings and part of many interventional guidelines for the treatment of manifest dementia. However, cognitive training (CT) on the other hand is far more domain specific and focuses on guided practice on a set of tasks that reflect particular cognitive functions. CT is thought to attenuate cognitive decline related to aging as well as disease-related decline [9, 10]. Further, limited evidence indicates that those beneficial effects remain observable even several years after intervention [11]. Positive effects were reported in multiple cognitive domains, including memory, reasoning, and speed of processing [11–13].

Crucially, as AD typically impacts episodic memory, semantic memory and visuospatial abilities [14–16], any CT against symptoms of AD should thus focus on these three domains and may also include training of working memory (WM). Adding a WM component to the training provides the possibility of far transfer benefits to other cognitive domains and utlimately to every-day life functioning [17].

#### Rationale of computerized cognitive training

Computerized cognitive training (CCT) has benefits over other types of CT as it readily lends itself to home-based use and individualized training protocols. For example, a study including 42 patients with amnestic mild cognitive impairment (MCI) found training using a tablet computer to robustly improve episodic memory function, while additionally enhancing visuospatial abilities [8]. Although direct comparisons are difficult, a current meta-analysis indicates that computer-based interventions outperforms therapist-led trainings in MCI [18]. Comparing CCT by content, training of multiple-domains outperformed single domain trainings [19]. Multi-component CCT has beneficial effects on a number of cognitive aspects such as global cognition, attention, working memory [11] as well as processing speed [12] and delayed recall [13].

The ultimate goal of CT, transferring its effects to activities of daily life or cognitive tasks regarding different cognitive domains (far transfer), is particularly difficult to achieve when the trained task is too narrowly designed. Theoretical frameworks for the investigation of transfer effects have been established [20]. Interestingly, when examining predictors of transfer from WM training, lower baseline performance predicted higher gains [21]. That same study indicated that old age was associated with lower gains on visuospatial WM tasks.

Discordant conclusions concerning optimal training protocols also affect CCT interventions [22]. Existing studies indicate that CCT for elderly individuals is best done in groups at an institution setting rather than at home to ensure good adherence i.a. due to social affiliation. A meta-analysis focusing on intervention characteristics, showed that CCT yields greater effect sizes when trained more than 3 times per week (*p* = 0.042), more than 8 total training weeks (*p* = 0.003) and more than 24 training sessions (*p* = 0.040) [19, 23]. However, reviews on the current state of research highlight the inconclusiveness on the best training protocols and the necessity of comprehensive and longitudinal assessments of a broad range of cognitive functions, including generalization of benefits from trained tasks to other cognitive activities (far transfer) [6].

Concluding from previous findings, we aim at specifically training episodic memory, semantic and spatial abilities and working memory while complying to recommendations for effective training protocols.

#### Neuroimaging

One crucial part to in-depth understanding of CCT effects and mechanisms is situated in its’ physiological and more specifically neuronal correlates. Several studies have combined cognitive training with magnetic resonance imaging (MRI) to investigate neuronal changes resulting from cognitive training [24, 25]. Imaging can be employed to demonstrate target engagement and underlying mechanisms by measuring brain changes over the intervention period [26]. Here, functional MRI (fMRI) can help to distinguish changes in the network underlying the task specifically trained (e.g., hippocampus in an episodic memory task) from changes in neuronal processing observable outside the task-specific network where they may play a compensatory role. Data on functional brain changes due to CT showed increased as well as decreased activity after the CT [27]. However, these differing findings are probably caused by the complex relationship between task performance and changes in brain activity. If increased brain activity after CT is associated with increased task performance, this might indicate successful compensatory brain activity [28]. Stable performance in combination with decreased brain activity after CT on the other hand might indicate more efficient task processing as a consequence of the CT.

In summary, possible task fMRI results indicating beneficial CT in older adults at risk of AD are:

I. Decrease in task-related brain network activity after CT and constant task performance as a consequence of more efficient task processing
II. Decreased activity in brain regions outside the task-related network because brain regions within the task-related network are more active again
III. Increased activity in previously hypoactive task-related brain network and increased task performance through CT (restoration)
IV. New activity in brain areas not active before CT (new compensatory activity) and increased task performance

Apart from task related fMRI, resting state fMRI (rsfMRI) provides valuable information about functional brain connectivity. This technique is often used to investigate structures like the default mode network (DMN), which consists of several brain areas particularly active during rest [29]. Another network often investigated in association with dementia is the central executive network (CEN) which is active during demanding tasks like working memory. Alterations within the DMN and CEN network have frequently been associated with dementia. In the context of AD for example, a decrease in the DMN connectivity has been reported repeatedly. Multi-domain CCTs were able to increase the connectivity between the hippocampus and frontal as well as the temporal brain areas during rest. Furthermore, connectivity within the DMN and CEN could be increased through a multi-domain cognitive training [10, 30].

Another useful technique to depict cerebral blood flow (CBF) is Arterial spin labelling (ASL), a non-invasive alternative to positron emission tomography. Several studies reported a pattern of hypoperfusion in MCI and dementia due to AD especially in the precuneus and posterior cingulate gyrus [31, 32]. Moreover, reduced blood flow in medial-temporal and parietal brain regions was associated with increased decline of functioning in daily life in healthy older adults or participants with MCI across 3 years [33]. Interestingly, cognitive training was able to increase global CBF in parieto-frontal regions in a sample of healthy older adults. This might indicate a possibility to reverse hypoperfusion, even in normal aging [34].

Apart from effects on functional brain activity, structural brain changes have been reported after CT as well. Nguyen et al. [27], for example, reported that CT may lead to increased grey matter volume or attenuate age related cortical thinning.

#### Blood measures

Blood measures are another way to estimate age or AD related brain changes. The presence of the Met-allele in the brain-derived neurotrophic factor (BDNF) genotype was associated with an decreased hippocampal volume in young adults [35] and an increased rate of cognitive decline in middle aged adults in episodic memory and executive functions across more than 6 years [36]. The apolipoprotein E (ApoE) 4 allele is a risk factor for AD. In the context of CT, it seems that ApoE non-carriers benefit more from a CT than carriers. But data about the moderating effect of ApoE on CT benefits is scarce and more studies are needed [37]. To estimate brain pathologies possibly indicating AD (e.g., amyloid plaques and tau fibrils), new blood plasma based measures of amyloid beta and phosphorylated Tau exists. These measures showed the ability to differentiate between amyloid PET positive and negative participants [38–40].

### Objectives

The main objective of this study is to train cognitive domains typically affected by AD through a serious game-based CCT and to assess the training effect on cognition and neural parameters of older adults in an at-risk or manifested stage of AD relative to an active and a waitlist control group. We set out to combine direct training of episodic memory, semantic, and spatial abilities with working memory training to facilitate transfer to every-day functioning. By combining group- and at-home based training elements, while integrating collaborative social elements both within the app as well as during the group sessions, we expect high adherence. In summary, the purpose of this study is 1) to investigate the effectiveness of our CCT on the three cognitive domains typically affected in AD, 2) investigate transfer effects and 3) to explore the neuronal underpinnings of the CCT.

We hypothesized that participants enrolled in the CCT group will show larger and more durable improvements in cognitive performance after training compared to those in either the active control group or the waitlist control group. We expect changes in functional brain activity to be observed during rest (i.e., increased connectivity in the DMN/CEN and increased CBF). Based on the findings reported in the previous section [10], we also expect increased functional connectivity between the hippocampus and frontal as well as temporal brain areas. In task fMRI, we expect indications for more efficient task-specific networks. When keeping the number of solved trials constant (e.g., in an externally paced task protocol) we expect declining activity due to higher task efficacy in the intervention group. In the self-paced SA task, we expect more solved trials in all participants but less activity increase in task-related networks after training compared to baseline activity. This finding would again reflect more efficient networks [28]. Furthermore, we aim to identify factors that moderate the magnitude of these effects. As shown in our previous cognitive training study [41], participants with less advantageous cognitive profiles at baseline, particularly, may benefit from cognitive interventions. Other factors which might moderate training benefits are blood and genetic markers like ApoE 4 genotypes and plasma amyloid which will also be analyzed during this study.

## Methods: participants, interventions and outcomes

### Setting, recruitment and eligibility criteria

Data collection will be conducted at the Interdisciplinary Memory Clinic (IDMC) in Bern and the Cantonal Hospital of Lucerne, Switzerland. Participants will be recruited by local newspaper adverts as well as directly through the respective Memory Clinic. A total of 162 elderly participants (*n* = 54 per study arm) from a continuum covering cognitively normal individuals at risk for AD with SCD to MCI (diagnosed or cut-off score in the cognitive telephone screening instrument (COGTEL) according to Alexopoulos [42]) and mild AD patients will be recruited. All participants will need to fulfill the following inclusion criteria: (1.) Ability to give their written consent to participate in the study; (2.) Age between 60 and 85 years; (3.) Native or fluent proficiency in German; (4.) Normal or corrected to normal vision and hearing; (5.) Ability to visit the study location repeatedly; (6.) MoCA (Montreal Cognitive Assessment [43] > 11. Exclusion criteria are patients with any contra-indications for MRI-scanning, substance abuse or severe medical conditions (of note: patients with mild depressive symptoms not fulfilling criteria for major depressive disorder are recruited into the sample to better represent clinical reality).

### Trial design and study setting

**Fig. 1:**
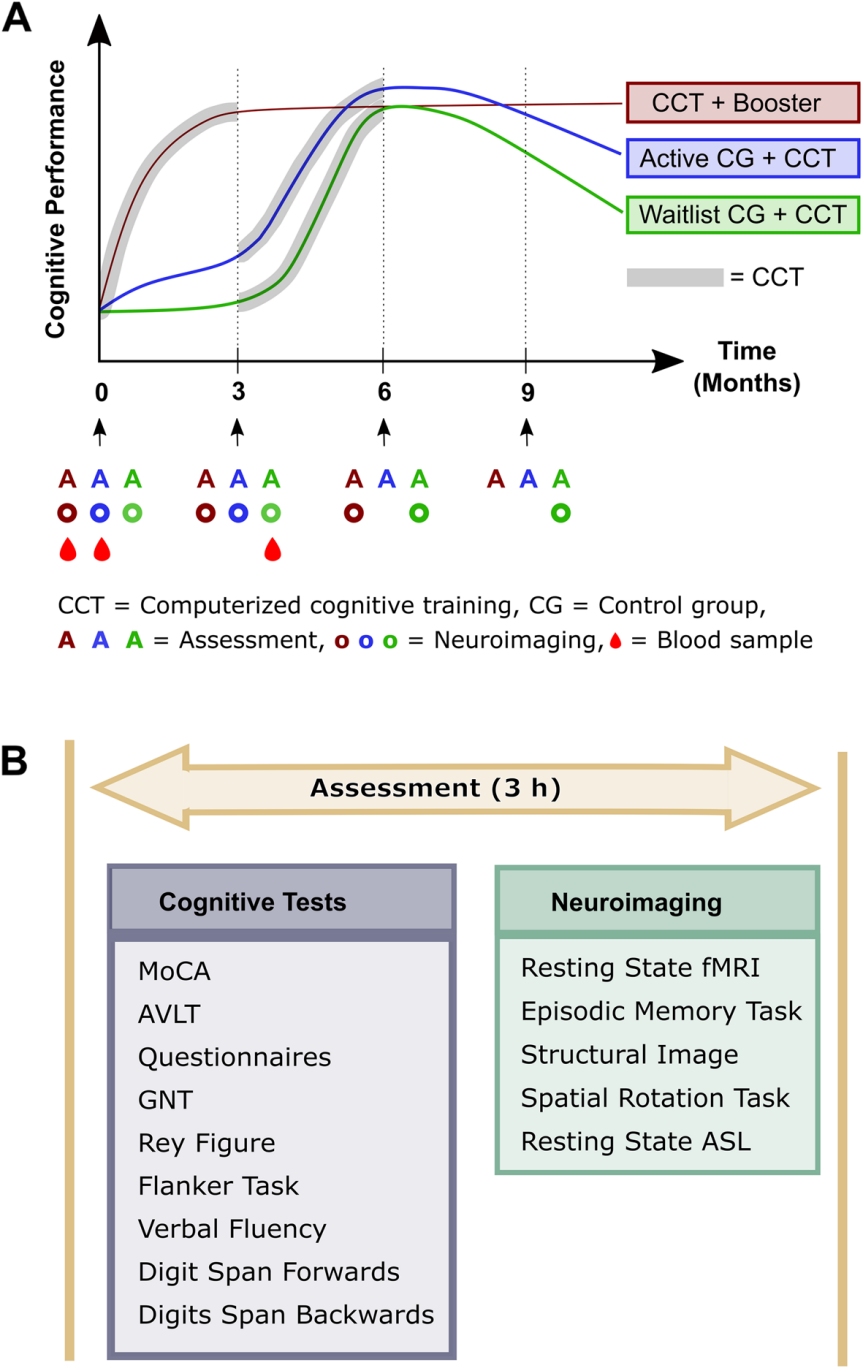
**a** Study Design: In the three study arms participants perform a cognitive assessment four times with an interval of 3 months in-between. Neuroimaging is performed at baseline and after 3 months in all study arms. After 6 months neuroimaging is performed in the cognitive training and the waitlist control group and after 9 months in the waitlist control group only. Blood samples are collected once, in the first group appointment. **b** Assessment: Each assessment has a duration of approximately 3 hours if neuroimaging is included. Abbreviations Figure a: CCT = Computerized cognitive training, CG = Control group, A = Assessment, o = Neuroimaging. Abbreviations Figure b: MoCA = Montreal Cognitive Assessment, AVLT = Auditory verbal learning test, GNT = Graded naming test, fMRI = Functional magnetic resonance Imaging, ASL = Arterial spin labelling

This study will be conducted in a bi-centric, randomized, placebo-controlled, within and partially blinded three arm design to investigate the effect of our CCT. The study design is presented in Fig. 1.

Participants will be randomly assigned to one of three study arms (training, active control, or waitlist control group) based on a stratified Mahalanobis distribution procedure conducted in R [44] balancing arms by level of cognitive impairment, age, and sex through a continuous adjustment of the randomization probabilities.

### Participant timeline

Participants will receive information that they will undergo a cognitively activating serious game-based computerized training starting either immediately or after a 3 months’ observation period but without knowing the distinction between the CCT and the active control group. Staff conducting the cognitive testing will be blinded to the study arm. The training will be conducted in small groups of 3–6 participants. The training group immediately starts with the CCT for 10 weeks, including weekly group sessions and will subsequently perform the training for twenty more weeks with monthly booster sessions. The waitlist control group will start the CCT with a delay of 10 weeks. The active control group will perform 10 weeks of unspecific cognitive activation (watching documentaries) before subsequently starting the CCT for 10 weeks. Ten weeks after the fourth assessment (i.e. after 3 × 10 weeks), all participants will be followed-up with a parallel version of the COGTEL.

### Intervention description

Based on literature and standardized cogntive tests, we designed four tablet-based training games for each of the three key cognitive domains specifically affected by AD (episodic memory, semantic memory and spatial abilites) in addition to WM-based training. All games are fully developed in-house, programmed in unity (http://unity3d. com/) and provided on 10.2 in. iPads. Individual games are connected by an overarching story-line (a sailboat traveling from one island to the next where each island represents a game) and are performed repeatedly within sessions. A detailed description of each game can be found in the supplementary material (see Additional file 1).

During most on-site group sessions (week 1–4, 6, and 8), two new training games are introduced and practiced with the support of the investigators. The games are distributed in an accumulative pseudo-randomized order to balance between memory components. Each game consists of different levels of difficulty and adapts automatically to the abilities of the participants. Furthermore, cooperative elements like a puzzle which can be solved as a group provides a social experience. According to recommendations of a recent meta-analysis, participants play the games daily for 24 minutes, i.e., three games for 8 minutes each [23]. The study design thus required between 50 and 150 days with training sessions depending on the study arm, resulting in each game being played at least 12 times. Before every at home training session participants will be asked to rate their motivation for the session and how they feel at the moment. After each session, they subjectively will rate their performance.

After 1 week of playing each game, we will assess game perception using the game perception questionnaire [45, 46]. General gaming experience is assessed with the core elements of gaming experience questionnaire (CEGEQ) [47].

### Explanation for the choice of comparators: active control group and waitlist control group

To test whether potential improvements in the intervention group result specifically from the training and not just from e.g. meeting as a group or performing an activating task, we use an active control group. To match the at-home element of the intervention group participants in the active control group will be asked to watch documentaries on the tablet computer matched for time and frequency of the CCT. To ensure that the participants are attentively watching the videos, pop-up windows with easy to solve multiple choice questions (e.g. “What is 2+3?”) randomly appear during the documentaries sessions. This active control condition aims to include the same level of social interaction in a group setting again matched for duration and frequency but without explicit cognitive training. To this end, participants are asked to discuss the documentaries with the other group members. Additionally, participants will play group games that are not cognitively demanding and where success is based on chance rather than skill (e.g., Bingo in slow speed). The active control intervention lasts 10 weeks. Afterwards, participants perform the same CCT as the intervention group.

Before staring the CCT, the wait-list control group will have a 10-week passive waiting period after the first cognitive assessment and MRI scan. The waitlist control group will allow us to quantify test-retest effects as well as habituation to the MRI scanner setting as in previous work by others [48].

### Strategies to improve adherence to the intervention and to promote participant retention

As previous CCT studies often faced mal-adherence, we included weekly groups sessions to not solely have a training purpose but to also encourage social interaction among participants. Additionally, based on research on the most useful intervention protocols, we encouraged daily training and followed up with booster sessions over 6 months for long-term benefits in the intervention group. Game characteristics favorable for training success such as a challenging but playful design were implemented based on previous research findings [49, 50]. To promote motivation and to provide direct assistance, participants are called after their first at-home training and a help button within the app triggers an automated e-mail to the investigator. Response data is transmitted to the investigator to track compliance to the training protocol and to provide low-threshold support in case of difficulties or potential non-adherence.

### MRI protocol

We will perform several MRI sequences before and after the CCT to assess functional and structural brain changes. Imaging data will help identify elements of restoration and compensation in task related brain activity after the CCT [51, 52]. Two fMRI tasks will be performed in this study. The first task will be an adapted version of a face-occupation task described in a previous study [53] to assess EM, while the second one is an adapted version of a task to assess spatial abilities (SA) [54]. In the face-occupation task, the association between face and occupation pairs has to be learned during the encoding phase. During the control condition, head contours are displayed and participants have to indicate, if the head contour belongs to a male or female. In the cued recall condition, the previously encoded faces are shown again and the question whether the occupation requires a university degree or an apprenticeship has to be answered. During the recognition condition, the faces are presented with two occupations and participants have to indicate which occupation is associated with the face. We expect activity in the medial temporal lobe and occipital brain areas as a previous study with a very similar paradigm showed activity in these brain areas. While the activity in the MTL is expected in EM tasks, the occipital activity might be caused through the task design, where different stimuli were used in the control than in the other conditions (i.e., pictures of faces vs head contours) [55]. In the SA task, participants are asked to mentally translate or rotate two puzzle pieces to indicate if they fit together to build a square. As control condition, two grey squares are presented in similarly shaped puzzle pieces. The participants are asked to indicate if both grey squares have exactly the same color. Based on previous results in the spatial abilities task, we expect activity in the superior parietal cortex bilaterally, the left inferior parietal cortex, bilateral lateral occipital cortex and the medial frontal gyrus. These brain areas are associated with complex visual-spatial construction tasks [54]. Please see the supplementary material for a detailed description of the fMRI tasks (see Additional file 2).

In the analysis of task fMRI, we will predefine regions of interest (i.e., regions with significant activity in the baseline assessment) and investigate how these areas change activity through the training. Additionally, we will also perform whole brain analyses to find new clusters of activity not present in the baseline assessment as a possible sign of compensatory activity [48]. Scanning at both study sites will be performed with 3 Tesla scanners (Bern: Siemens Magnetom Prisma, 32 channel head coil; Lucerne: Siemens Magnetom Vida, 64 channel head coil). From all participants, T1- weighted images (MP2RAGE, TR = 5000 ms, TE = 2.98 ms, TI 1/2 = 700 ms/2500 ms, flip angle 1/2 = 4 degrees/5 degrees, FOV = 256, matrix = 256 × 256, voxel size = 1 × 1 × 1 mm, 176 slices) will be acquired. For the fMRI sequences we will acquire echo-planar imaging (EPI) with 360 (resting state fMRI), 604 (SA Task) and 638 (EM task) volumes (TR = 1000 ms, TE = 37 ms, flip angle = 30 degree, FoV = 230 mm, matrix = 92 × 92, accelerating factor 8, voxel size 2.5 × 2.5 × 2.5 mm, 72 slices). Furthermore, we will perform an ASL sequence (91 volumes, TR = 3300 ms, TE = 13 ms, Flip angle = 90 degree, FoV = 230 mm, matrix = 64 × 64, voxel size 3.6 × 3.6 × 6.0 mm, 22 slices).

### Blood analysis

Blood samples will be taken once during the first group session. Genotyping for BDNF ApoE will be performed. Furthermore, plasma pTau181, Amyloid Beta_42_, Amyloid Beta_40_, glial fibrillary acidic protein and neurofilament light will be analyzed to estimate AD related brain pathology [38–40].

### Outcomes

Each participant will perform the cognitive assessments four times in intervals of approximately 10 weeks. The individual cognitive profile will be assessed through a set of paper-pencil and tablet-based cognitive tests designed to evaluate typical AD components. More precisely, the general cognitive performance will be assessed in a paper-pencil version of the MoCA. The following tests will be administered as tablet-based versions: the auditory verbal learning test (AVLT) [56] to assess episodic memory, the Rey-Osterrieth Complex Figure Test (ROCF) [57] for spatial abilities/memory, and verbal fluency as well as the Graded Naming Task (GNT-30) [58] will be used to probe semantic memory. Additionally, participants will performed the Flanker Test [59] to assess attention/response inhibition abilities and the digit span test (forward and backwards) as measure of short-term memory and working memory respectively. Parallel versions of the tests will be used whenever possible. Based on this comprehensive test battery, a cognitive composite score will be calculated as primary outcome of cognitive performance.

Moreover, participants will be asked to complete questionnaires to assess their form of the day, their situational motivation [60], quality of life [61] and depressive symptoms trough the geriatric depression scale (GDS) [62]. Through in-house designed or adapted questionnaires, participants’ expectancy towards the cognitive training (Additional file 3) [63], and subjective cognitive performance (Additional file 4) will be assessed. Subjective cognitive performance will additionally be assessed in an informant rated version of the questionnaire (filled out by a close friend or relative) (Additional file 5). Unexperienced participants will have the opportunity to get used to the tablet and to have one on one support from the investigator before the training starts.

The mentioned tests will be supplemented by a set of paper-pencil based questionnaires that will be sent to the participants before the first on-site meeting. These preliminary questionnaires asses activities of daily living [64], handedness [65], and the informant rated subjective memory performance questionnaire.

### Statistical methods

#### Sample size

For our primary analysis (“Does change in performance on the three typical AD domains 3 months after the start of the training differ between groups?”), a repeated- measures ANOVA is planned (3 groups with 2 time points). We computed the necessary sample size using G*Power [66] to ensure a power of 80% to detect an assumed moderate effect (i.e. effect size: η2 = 0.06, f = 0.25) with α of 5%. The power analysis revealed a total sample size of 162 (54 participants per group). Moreover, we performed a power analysis for testing the interaction between the groups and all four time points within a repeated-measures ANOVA using the same parameters as before (α = 5%, power = 80%, η2 = 0.06) as well as a non-sphericity correction factor of 0.75. According to this power analysis a total sample size of 96 (32 participants per group) is needed.

#### Statistical methods for primary and secondary outcomes

Primary outcomes are three composite scores corresponding to the domains mostly affected by AD. One advantage of composite scores is a reduced number of statistical tests. At the same time, separate composite scores for the three domains allow to assess and compare changes in the scores individually. For each of the domains, three variables will be included in the composite score (EM: AVLT learning sum, immediate and delayed recall; SA: ROCF encoding, immediate and delayed recall; semantics: semantic fluency from the MoCA, phonemic fluency and GNT). We will perform a principal component analysis (PCA) for each of the respective domains.

All scores will be scaled and centralized on the scores from the baseline assessment and weighted if appropriate according to their loadings in the PCA. The main hypothesis will be tested with a repeated-measures ANOVA for which an adequate level of power should be guaranteed (according to the power analysis described before). Further analyses (using all time points and additional numeric subject characteristics as predictor variables) will be performed with linear mixed models as they allow for more sophisticated model design and a better handling of missing data. As covariates, the factors study site and study arm will be included in all analyses. Neuroimaging data will be analyzed with SPM12 and related toolboxes (e.g., Functional Connectivity (CONN) toolbox [67]). Apart from task related fMRI, resting state fMRI, volumetrical images and ASL will be performed.

To assure meaningful data, non-adherence to the training regimen leads to exclusion of further analysis. Non-adherence is defined as completing less than 50% of the required training sessions and attendance of less than 70% of the group sessions.

Given that a relatively high number of games are purposely built for this study, we expect the need for continuous adjustments based on feedback from participants. Should substantial adjustments be necessary, we will introduce release number as a covariate in the analysis.

#### Dissemination of outcomes

We will disseminate findings from this research as widely as possible to reach academic, clinical and public audiences. All academic publications will be peer-reviewed, open access and we will share findings through a range of other channels, e.g. newsletters, study websites, social media, presentations at conferences and events.

#### Adverse event reporting and study integrity

Health hazards will be reported to the Sponsor-Investigator within 24 hours upon becoming aware of the event. Health hazards will be reported to the local Ethics Committee within 2 days.

Substantial changes to the study setup and study organization, the protocol and relevant study documents are submitted to the local Ethics Committee for approval before implementation.

## Discussion

As main goal, the present RCT focusses on the effectiveness of CCT protocols as well as their neuronal underpinnings while ensuring high adherence. From a scientific viewpoint, our study will provide new insights how well a CCT can improve performance on the targeted cognitive domains. Even relatively mild effects of CCT would have a substantial economic impact given to possibility of a wide implementation. Models established for the UK indicate that a symptomatic intervention which leads to a one-point increase in the mini-mental state examination (MMSE) [68] would reduce health costs by millions if applied early in the disease process even if the effects of the intervention would wear out completely over 3 years [7].

The study overcomes major short-comings of prior research projects: First, the design of the study combines individual at home training with the social aspect of group meetings, leading to high adherence due to the social affiliation. Moreover, having multiple cognitive assessments and follow-ups over a one-year period allows analysis of longitudinal benefits of the training (e.g., duration of possible CCT effects). Additionally, the intervention group receiving a daily training over 9 months will help to assess possible dose-response relationships. Loss of motivation and thus declining training adherence was one of the major shortcoming of previous studies. We addressed this through the appealing design of the games, specifically tailored to please older players. Also, the interplay of at home training and repeated and frequent group meetings is thought to play a crucial role in keeping regimen adherence and motivation high.

Additionally, the study implements recommendations [9] for further RCTs conducted on this matter, e.g., by applying treatments to a rather heterogeneous sample (e.g., with respect to age range and wide range of symptom severity), using both an active as well as in waitlist control group, adding behavioural and biological markers, combining self-assessments and clinical assessments as outcome variables and following standardized protocols. Beyond this, we added follow-up visits after study completion to gain insight into long-term effects of our interventions that are both clinically and economically relevant.

Nevertheless, this study faces some methodological limitations and other challenges. First, given the nature of the study design, neither blinding of the participants nor the group leaders can be achieved. However, all staff conducting the cognitive testing and/or the MRI scanning are blinded to the study arm. Furthermore, the games were purposely programmed for this study. Despite piloting, we expect the need for continuous adjustments to the games.

Thanks to MRI data and the blood-based biomarkers, this RCT will add relevant data regarding physiological underpinnings of cognitive training. Crucially, due to combination of behavioural, cognitive and physiological data, our RCT will have a high predictive value on training success and in turn on the individualization of cognitive training protocols. From a clinical perspective, scientifically based prediction schemes will be critical to the development for the most effective treatments and thus for effective cognitive interventions tailored to the individual.

To conclude, this study sets out to provide high-quality longitudinal results with appropriately selected outcome measures to determine whether CCT can contribute to maintaining cognitive function and eventually real-world daily functioning.

### Trial status

This RCT was registered 1st of July 2020 at clinicaltrials.gov (Identifier NCT04452864). This is the first version of the protocol. Recruitment started in October 2020 and is ongoing.

## Supplementary Information

**Additional file 1. ** Description of the cognitive training app.

**Additional file 2. ** Functional MRI tasks.

**Additional file 3. ** Expectations towards cognitive training questionnaire.

**Additional file 4. ** Subjective cognitive performance questionnaire (Self-assessment).

**Additional file 5. ** Subjective cognitive performance questionnaire (Informant rated).

## Data Availability

Data relevant for publications will be published on a suitable repository (e.g., Zenodo). Pseudomized data will be stored and distributed by an on premises GitLab instance hosted on the virtual infrastructure of the University of Bern with external object storage provided by SWITCH. On all datasets, documents and biological samples participants are only identified by a unique participant number. The raw data are available on request from the corresponding author, EB.

## Abbreviations

AD: Alzheimer’s Disease
ApoE: Apolipoprotein E
ASL: Arterial spin labelling
AVLT: Auditory verbal learning test
BDNF: Brain derived neurotrophic factor
CBF: Cerebral blood flow
CCT: Computerized cognitive training
CEN: Central executive network
CG: Control Group
CT: Cognitive training
DLPFC: Dorsolateral prefrontal cortex
DMN: Default mode network
EM: Episodic memory
EPI: Echo-planar imaging
fMRI: Functional magnetic resonance imaging
GNT-30: Graded Naming Test
HDL: High-density lipoprotein
IDMC: Interdisciplinary Memory Clinic
MCI: Mild cognitive impairment
MoCA: Montreal Cognitive Assessment
MRI: Magnetic resonance imaging
rsfMRI: Resting state functional magnetic resonance imaging
RCT: Randomized controlled trial
ROCF: Rey-Osterrieth complex figure test
SA: Spatial abilities
SCD: Subjective cognitive decline
SM: Semantic memory
SR: Spatial Rotation
WCG: Waitlist control group
WM: Working memory

## References

[1] Peter J, Abdulkadir A, Kaller C, Kümmerer D, Hüll M, Vach W (2014). Subgroups of Alzheimer’s disease: stability of empirical clusters over time. J Alzheimers Dis.

[2] McKhann GM, Knopman DS, Chertkow H, Hyman BT, Jack CR, Kawas CH (2011). The diagnosis of dementia due to Alzheimer’s disease: recommendations from the National Institute on Aging-Alzheimer’s association workgroups on diagnostic guidelines for Alzheimer’s disease. Alzheimers Dement.

[3] Alzheimer’s Association (2021). Alzheimer’s disease: facts and figures special report race, ethnicity and Alzheimer’s in America. Alzheimers Dement.

[4] Pike KE, Cavuoto MG, Li L, Wright BJ, Kinsella GJ (2021). Subjective cognitive decline: level of risk for future dementia and mild cognitive impairment, a meta-analysis of longitudinal studies. neuropsychology review.

[5] Koppara A, Wagner M, Lange C, Ernst A, Wiese B, König HH (2015). Cognitive performance before and after the onset of subjective cognitivedecline in old age. Alzheimer’s Dement Diagnosis, Assess Dis Monit.

[6] Hao L, Xing Y, Li X, Mu B, Zhao W, Wang G (2019). Risk factors and neuropsychological assessments of subjective cognitive decline (plus) in Chinese memory clinic. Front Neurosci.

[7] Barnett JH, Lewis L, Blackwell AD, Taylor M (2014). Early intervention in Alzheimer’s disease: a health economic study of the effects of diagnostic timing. BMC Neurol.

[8] Savulich G, Piercy T, Fox C, Suckling J, Rowe JB, O’brien JT (2017). Cognitive training using a novel memory game on an iPad in patients with amnestic mild cognitive impairment (aMCI). Int J Neuropsychopharmacol.

[9] Gates NJ, Vernooij RWM, Di Nisio M, Karim S, March E, Martínez G, et al. Computerised cognitive training for preventing dementia in people with mild cognitive impairment. Cochrane Database Syst Rev. 2019;(3). Art. No.: CD012279. 10.1002/14651858.CD012279.pub2 PMC6415132 30864747

[10] Ten Brinke LF, Davis JC, Barha CK, Liu-Ambrose T (2017). Effects of computerized cognitive training on neuroimaging outcomes in older adults: a systematic review. BMC Geriatr.

[11] Hill NTM, Mowszowski L, Naismith SL, Chadwick VL, Valenzuela M, Lampit A (2017). Computerized cognitive training in older adults with mild cognitive impairment or dementia: a systematic review and meta-analysis. Am J Psychiatry.

[12] Rodella C, Bernini S, Panzarasa S, Sinforiani E, Picascia M, Quaglini S (2022). A double-blind randomized controlled trial combining cognitive training (CoRe) and neurostimulation (tDCS) in the early stages of cognitive impairment. Aging Clin Exp Res.

[13] Nousia A, Martzoukou M, Siokas V, Aretouli E, Aloizou AM, Folia V (2021). Beneficial effect of computer-based multidomain cognitive training in patients with mild cognitive impairment. Appl Neuropsychol.

[14] Dudas RB, Clague F, Thompson SA, Graham KS, Hodges JR (2005). Episodic and semantic memory in mild cognitive impairment. Neuropsychologia.

[15] Quental NBM, Brucki SMD, Bueno OFA (2009). Funções visoespaciais na doença de alzheimer de intensidade leve: Estudo preliminar. Dement e Neuropsychol.

[16] Rubiño J, Andrés P (2018). The face-name associative memory test as a tool for early diagnosis of alzheimer’s disease. Front Psychol.

[17] Karbach J, Verhaeghen P (2014). Making working memory work: a Meta-analysis of executive-control and working memory training in older adults. Psychol Sci.

[18] Chandler MJ, Parks AC, Marsiske M, Rotblatt LJ, Smith GE (2016). Everyday impact of cognitive interventions in mild cognitive impairment: a systematic review and Meta-analysis. Neuropsychol Rev.

[19] Bahar-Fuchs A, Martyr A, Goh A, Sabates CL (2019). Cognitive rehabilitation for people with mild to moderate dementia. Cochrane Database Syst Rev..

[20] Noack H, Lövdén M, Schmiedek F (2014). On the validity and generality of transfer effects in cognitive training research. Psychol Res.

[21] Zinke K, Zeintl M, Rose NS, Putzmann J, Pydde A, Kliegel M (2014). Working memory training and transfer in older adults: effects of age, baseline performance, and training gains. Dev Psychol.

[22] Anderson-Hanley C, Barcelos NM, Zimmerman EA, Gillen RW, Dunnam M, Cohen BD (2018). The aerobic and cognitive exercise study (ACES) for community-dwelling older adults with or at-risk for mild cognitive impairment (MCI): neuropsychological, neurobiological and neuroimaging outcomes of a randomized clinical trial. Front Aging Neurosci..

[23] Chiu H-L, Chu H, Tsai J-C, Liu D, Chen Y-R, Yang H-L (2017). The effect of cognitive-based training for the healthy older people: a meta-analysis of randomized controlled trials. PLoS One.

[24] Miotto EC, Batista AX, Simon SS, Hampstead BM (2018). Neurophysiologic and cognitive changes arising from cognitive training interventions in persons with mild cognitive impairment: a systematic review. Neural Plast..

[25] Hosseini SMH, Kramer JH, Kesler SR (2014). Neural correlates of cognitive intervention in persons at risk of developing alzheimer’s disease. Front Aging Neurosci.

[26] Vemuri P, Fields J, Peter J, Klöppel S (2016). Cognitive interventions in Alzheimer’s and Parkinson’s diseases: emerging mechanisms and role of imaging. Curr Opin Neurol.

[27] Nguyen L, Murphy K, Andrews G (2019). Cognitive and neural plasticity in old age: A systematic review of evidence from executive functions cognitive training. Ageing Res Rev.

[28] Stern Y (2009). Cognitive reserve. Neuropsychologia.

[29] Hafkemeijer A, van der Grond J, Rombouts SARB (2012). Imaging the default mode network in aging and dementia. Biochim Biophys Acta Mol basis Dis.

[30] Cao W, Cao X, Hou C, Li T, Cheng Y, Jiang L (2016). Effects of cognitive training on resting-state functional connectivity of default mode, salience, and central executive networks. Front Aging Neurosci..

[31] Thomas B, Sheelakumari R, Kannath S, Sarma S, Menon RN (2019). Regional cerebral blood flow in the posterior cingulate and precuneus and the entorhinal cortical atrophy score differentiate mild cognitive impairment and dementia due to Alzheimer disease. Am J Neuroradiol.

[32] Tosun D, Schuff N, Mathis CA, Jagust W, Weiner MW (2011). Spatial patterns of brain amyloid-β burden and atrophy rate associations in mild cognitive impairment. Brain.

[33] Catasús CAS, Willemsen A, Boellaard R, Juarez-Orozco LE, Samper-Noa J, Aguila-Ruiz A (2018). Episodic memory in mild cognitive impairment inversely correlates with the global modularity of the cerebral blood flow network. Psychiatry Res Neuroimaging.

[34] Chapman SB, Aslan S, Spence JS, Hart JJ, Bartz EK, Didehbani N (2015). Neural mechanisms of brain plasticity with complex cognitive training in healthy seniors. Cereb Cortex.

[35] Bueller JA, Aftab M, Sen S, Gomez-Hassan D, Burmeister M, Zubieta JK (2006). BDNF Val66Met allele is associated with reduced hippocampal volume in healthy subjects. Biol Psychiatry.

[36] Boots EA, Schultz SA, Clark LR, Racine AM, Darst BF, Koscik RL (2017). BDNF Val66Met predicts cognitive decline in the Wisconsin registry for Alzheimer’s prevention. Neurology.

[37] Roheger M, Meyer J, Kessler J, Kalbe E (2020). Predicting short- and long-term cognitive training success in healthy older adults: who benefits?. Aging Neuropsychol Cogn.

[38] Thijssen EH, La Joie R, Wolf A, Strom A, Wang P, Iaccarino L (2020). Diagnostic value of plasma phosphorylated tau181 in Alzheimer’s disease and frontotemporal lobar degeneration. Nat Med.

[39] Verberk IMW, Thijssen E, Koelewijn J, Mauroo K, Vanbrabant J, De Wilde A (2020). Combination of plasma amyloid beta (1-42/1-40) and glial fibrillary acidic protein strongly associates with cerebral amyloid pathology. Alzheimers Res Ther.

[40] Verberk IMW, Slot RE, Verfaillie SCJ, Heijst H, Prins ND, van Berckel BNM (2018). Plasma amyloid as Prescreener for the earliest Alzheimer pathological changes. Ann Neurol.

[41] Krebs C, Peter J, Wyss P, Brem A-K, Klöppel S. Transcranial electrical stimulation improves cognitive training effects in healthy elderly adults with low cognitive performance. Clin Neurophysiol. 2021;(xxxx). 10.1016/j.clinph.2021.01.034. 10.1016/j.clinph.2021.01.034 33875372

[42] Alexopoulos P, Skondra M, Kontogianni E, Vratsista A, Frounta M, Konstantopoulou G (2021). Validation of the cognitive telephone screening instruments COGTEL and COGTEL+ in identifying clinically diagnosed neurocognitive disorder due to Alzheimer’s disease in a naturalistic clinical setting. J Alzheimers Dis.

[43] Nasreddine ZS, Phillips NA, Bédirian V, Charbonneau S, Whitehead V, Collin I (2005). The Montreal cognitive assessment, MoCA: a brief screening tool for mild cognitive impairment. J Am Geriatr Soc.

[44] RStudio Team. RStudio Team. RStudio: Integrated Development for R; RStudio, PBC, Boston, MA; 2020. Available from: http://www.rstudio.com/.

[45] Chesham A, Gerber SM, Schütz N, Saner H, Gutbrod K, Müri RM, et al. Search and match task: development of a taskified match-3 puzzle game to assess and practice visual search. JMIR Serious Games. 2019;7(2). 10.2196/13620 PMC6532342 31094325

[46] Boot WR, Champion M, Blakely DP, Wright T, Souders DJ, Charness N (2013). Video games as a means to reduce age-related cognitive decline: attitudes, compliance, and effectiveness. Front Psychol.

[47] Calvillo-gámez EH, Cairns P, Cox AL (2010). Evaluating user experience in games.

[48] Belleville S, Clément F, Mellah S, Gilbert B, Fontaine F, Gauthier S (2011). Training-related brain plasticity in subjects at risk of developing Alzheimer’s disease. Brain.

[49] Diaz-Orueta U, Facal D, Nap HH, Ranga M-M (2012). What is the key for older people to show interest in playing digital learning games? Initial qualitative findings from the LEAGE project on a multicultural European sample. Games Health J.

[50] De Schutter B (2011). Never too old to play: the appeal of digital games to an older audience. Games Cult.

[51] Scheller E, Minkova L, Leitner M, Klöppel S (2014). Attempted and successful compensation in preclinical and early manifest neurodegeneration - a review of task fMRI studies. Front. Psychiatry.

[52] Krebs C, Peter J, Klöppel S (2018). Wissenschaftlich begründetes Gedächtnistraining bei kognitiver Störung. Swiss Arch Neurol Psychiatry Psychother.

[53] Klink K, Peter J, Wyss P, Klöppel S (2020). Transcranial electric current stimulation during associative memory encoding: comparing tACS and tDCS effects in healthy aging. Front Aging Neurosci.

[54] Seydell-Greenwald A, Ferrara K, Chambers CE, Newport EL, Landau B (2017). Bilateral parietal activations for complex visual-spatial functions: evidence from a visual-spatial construction task. Neuropsychologia.

[55] De Quervain DJF, Papassotiropoulos A (2006). Identification of a genetic cluster influencing memory performance and hippocampal activity in humans. Proc Natl Acad Sci U S A.

[56] Bean J, Kreutzer JS, DeLuca J, Caplan B (2011). Rey Auditory Verbal Learning Test, Rey AVLT. Encyclopedia of clinical neuropsychology.

[57] Osterrieth PA (1944). Le test de copie d’une figure complexe; contribution à l’étude de la perception et de la mémoire. [Test of copying a complex figure; contribution to the study of perception and memory.]. Arch Psychol (Geneve).

[58] Williams BW, Mack W, Henderson VW (1989). Boston naming test in Alzheimer’s disease. Neuropsychologia.

[59] Zelazo PD, Anderson JE, Richler J, Wallner-Allen K, Beaumont JL, Conway KP (2014). NIH toolbox cognition battery (CB): validation of executive function measures in adults. J Int Neuropsychol Soc.

[60] Guay F, Vallerand RJ, Blanchard C (2000). On the assessment of situational intrinsic and extrinsic motivation: the situational motivation scale (SIMS). Motiv Emot.

[61] Stevanovic D (2011). Quality of life enjoyment and satisfaction questionnaire - short form for quality of life assessments in clinical practice: a psychometric study. J Psychiatr Ment Health Nurs.

[62] Yesavage JA, Brink TL, Rose TL, Virwnia H, Adfy M, Leirer VO (1983). Development and validation of a geriatric depression screening scale: Preliminary report. Vol. 17, J. psychial. Rex.

[63] Devilly GJ, Borkovec TD. Psychometric properties of the credibility/expectancy questionnaire. J Behav Ther Exp Psychiatry. 2000;31(2):73–86. 10.1016/s0005-7916(00)00012-4 11132119

[64] Graf C (2008). Instrumental activities of daily living scale. Am J Nurs..

[65] Oldfield RC (1971). The assessment and analysis of handedness: the Edinburgh inventory. Neuropsychologia.

[66] Faul F, Erdfelder E, Lang A-G, Buchner A (2007). G*power 3: a flexible statistical power analysis program for the social, behavioral, and biomedical. Behav Res Methods.

[67] Whitfield-Gabrieli S, Nieto-Castanon A (2012). Conn: a functional connectivity toolbox for correlated and Anticorrelated brain networks. Brain Connect.

[68] Arevalo-Rodriguez I, Smailagic N, Roquéi Figuls M, Ciapponi A, Sanchez-Perez E, Giannakou A (2015). Mini-mental state examination (MMSE) for the detection of Alzheimer’s disease and other dementias in people with mild cognitive impairment (MCI). Cochrane Database Syst Rev..

[69] Chan AW, Tetzlaff JM, Altman DG, Laupacis A, Gøtzsche PC, Krleža-Jerić K (2013). Spirit 2013 statement: defining standard protocol items for clinical trials. Chinese J Evidence-Based Med.

