# Supplementary material for: Can a serious game-based cognitive training attenuate cognitive decline related to Alzheimer’s disease? Protocol for a randomized controlled trial
Source: BMC Psychiatry. 2022 Aug 12;22:552. doi: 10.1186/s12888-022-04131-7 (PMC9373273; doi:10.1186/s12888-022-04131-7)
Supplement: Supplementary file 1 — Additional file 1. Description of the cognitive training app. [file 12888_2022_4131_MOESM1_ESM.docx]

# **Additional file 1.**

# Disclaimer: We hold copyright of all pictures used in the figures of this document.

# **Description of the cognitive training app**

The main menu of the CCT app is composed of a group of islands of which each island represents a training game. A depiction of the main menu can be found in figure 1. The player has a spirit animal with which he travels from one island to the next to complete the training. The spirit animal can be chosen by the player (initially either a cat or a dog). As small individual achievements, new spirit animals unlock throughout the training once the player has reached a predefined goal (e.g. reaching a certain difficulty level or completing a certain amount of training sessions). These goals are unknown to the player. During each training session, a set of three games has to be played to complete the session. Games are distributed in a pseudo-randomized order within sessions in order to ensure all games and thus cognitive components are equally trained. During each game, a timer indicating the duration of the session appears in the upper right corner of the tablet. Furthermore, a puzzle was which can be solved as a training group through to completion of training sessions was implemented in order to provide a social experience even during the at-home sessions.


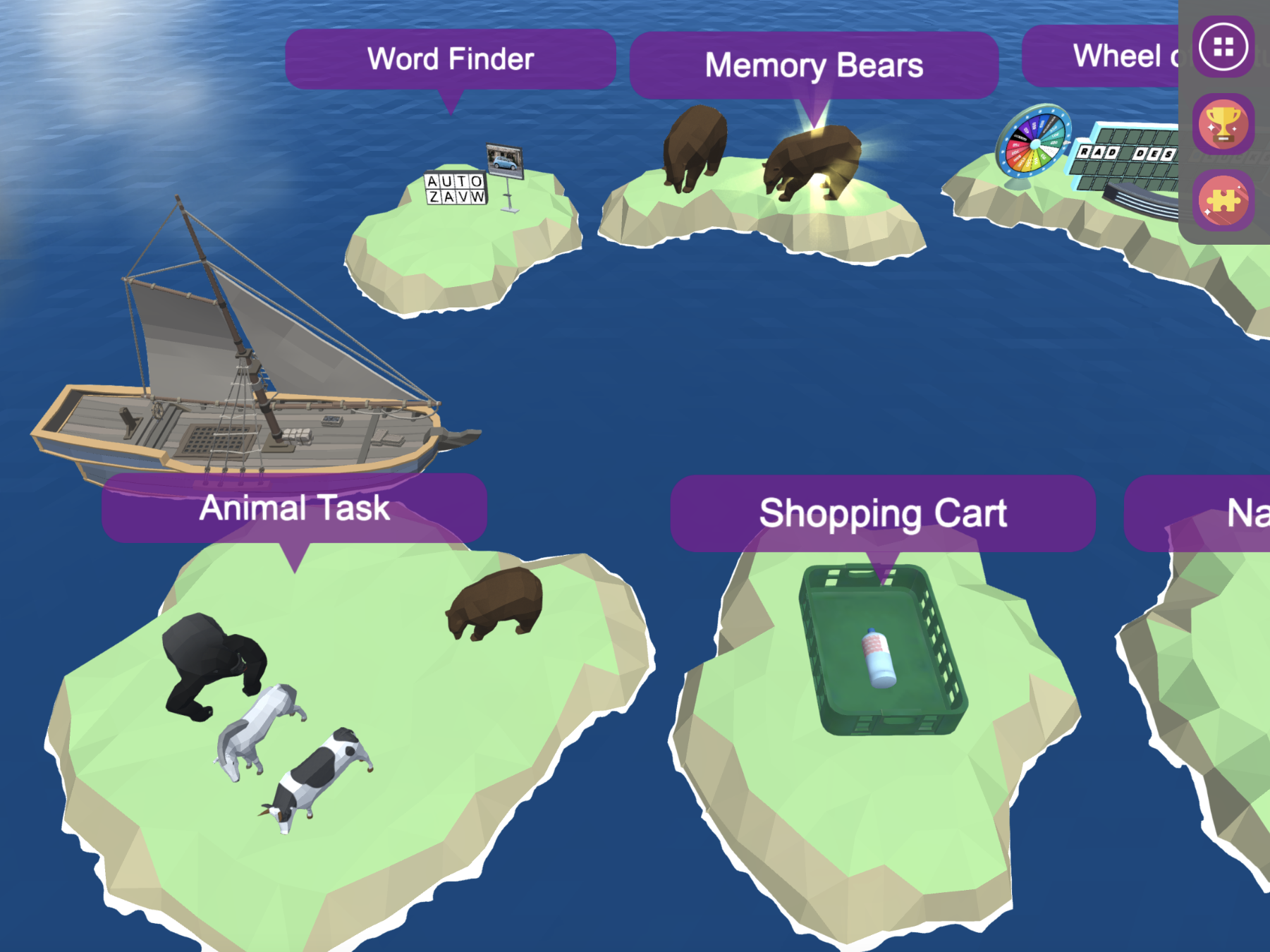


*Figure 1: Main menu of the training app. A sailboat traveling from one island to the next symbolizes the player’s progress in one training session. Each island represents a training game.*

# **Description of tablet-based serious games**

The present CCT aims at specifically training cognitive domains typically affected by AD (i.e., episodic memory, spatial abilites, semantic memory). We also include working memory training games in order to facilitate transfer of the training effects. However, due to the design of the study and the interconnectedness of memory components each training game is simulatenously training a range of different cognitive funtions. We thus determined expected loadings of the training effects onto different cognitive functions. Table 1 is an overview of the CCT games and their respective loading on each trained memory domains.

|  | **Working Memory** | **Episodic Memory** | **Spatial Abilities** | **Semantic Memory** |
| --- | --- | --- | --- | --- |
| **Memory Bears** | +++ | - | ++ | - |
| **Animal Park** | +++ | - | + | - |
| **Safari** | +++ | - | - | + |
| **Quiz Game** | +++ | + | - | ++ |
| **What's my name?** | + | +++ | - | - |
| **Billboards** | + | +++ | + | + |
| **Shopping basket** | + | ++ | - | + |
| **Artificial language learning** | + | +++ | - | + |
| **Mental Map** | ++ | - | +++ | - |
| **Magic Camera** | + | - | +++ | - |
| **Wine Cellar** | + | - | +++ | - |
| **Mansion Game** | + | ++ | +++ | - |
| **Word Grid** | + | - | + | +++ |
| **Pun Game** | + | - | - | +++ |
| **Famous Faces** | - | - | - | +++ |
| **Wheel of Fortune** | + | - | - | +++ |

*Table 1: Overview of our CCT games and their respective loadings on working memory, episodic memory, spatial abilites and semantic memory on a scale from 0 to 3 ( “-“ indicating no expected loading; “+++” indicating the highest possible loading)*

## **Episodic Memory**

Deficits in episodic memory predominate all stages of Alzheimer’s Disease (AD) including its prodromal phase of amnestic mild cognitive impairment (MCI) with deficits in e.g., person naming and item recognition (1,2). Also in normal aging, episodic memory is typically the first memory component to decline. As a classic episodic memory component, the ability to recall previously encountered information is impaired in all stages of AD pathology (3,4). Not only delayed recall but also the encoding of information is weakened, which additionally hinders subsequent recall (4). Additionally, human faces are critical socio-psychological signals in daily life. Impairment in face-name memory is a significant indicator of impairment in episodic memory and an early sign of AD. Remembering the association of faces-name pairings are highly associated with episodic memory as well as additionally challenging semantic memory performance. Face-name memory requires a sophisticated cognitive process not only because of the arbitrary association of faces and names but also because of the similarity and complexity among faces (5). Face-name training has been shown to be effectful in early AD patients, with training-related gains being maintained up to six months (6–8).

### **Billboards**

“Billboards” takes place in a artifical city scenery. Here, participants see a series of billboards whilst being driven through a city by car. An initial encoding phase is followed by two recall phases. In the first recall phase, participants are asked which of two billboards was shown first in the sequence. Then, the car takes another tour through the city and new billboards are displayed. Again, participants rate which billboard was presented first. After this second tour, the participant has to reproduce the order of billboards during the *first* tour. The stimuli of both tours are thematically different (animals and buildings) to better distinguish the rounds. “Billboards” is presented in figure 2.


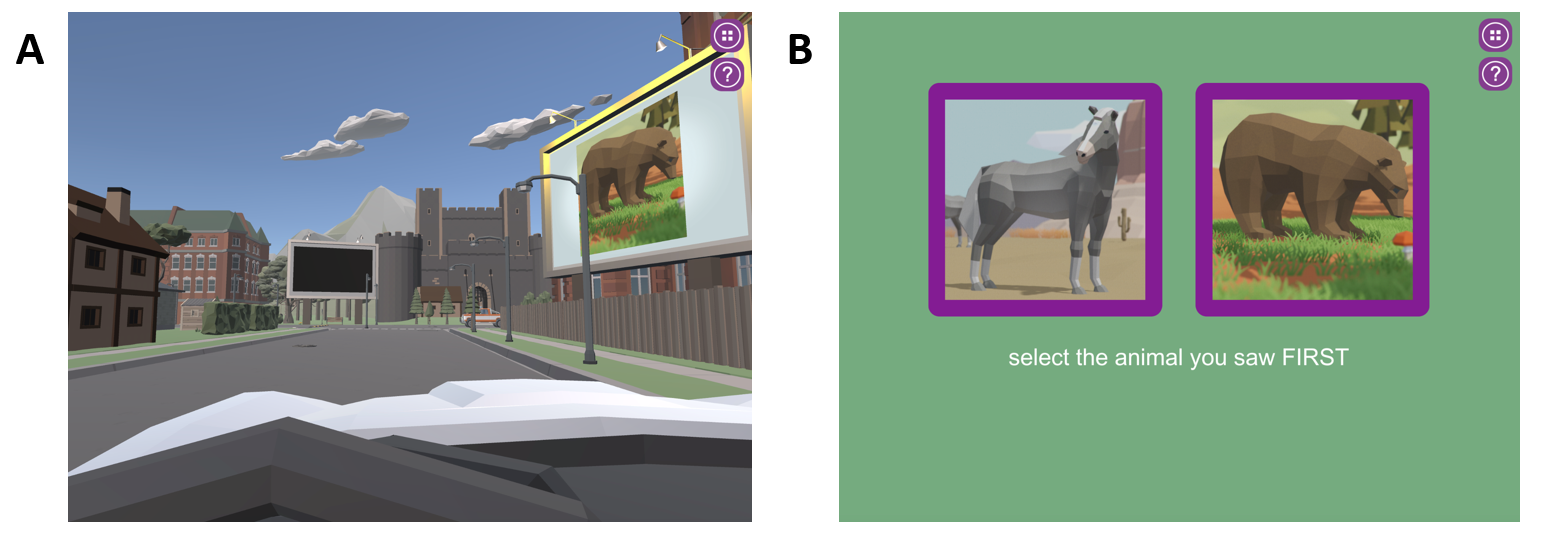


*Figure 2: Billboards. A) Encoding phase: car driving through the artificially build city. Billboards are depicted on the side of the road. B) Recall phase: Multiple choice of possible answers.*

### **What’s my name?**

“What’s my name?” mimicks the real-life situation of meeting new people and is inspired by Robert and collegues (9). Several photographs of people are presented and participants are asked to memorize the faces and associated names. Initially, two photographs are presented and the number increases with higher levels of difficulty.

After learning the associations, the names disappear and two of the previously seen faces are shown. Then, the player has to remember their names and has to hand objects to the correct person by dragging and dropping them to the respective person. Finally, the player sees all the previously encoded persons and has to drag and drop nametags to the correct person. The face and name associations are fixed and do not change throughout the game to more realistically depict an every-day life situation. “What’s my name” is presented in figure 3.


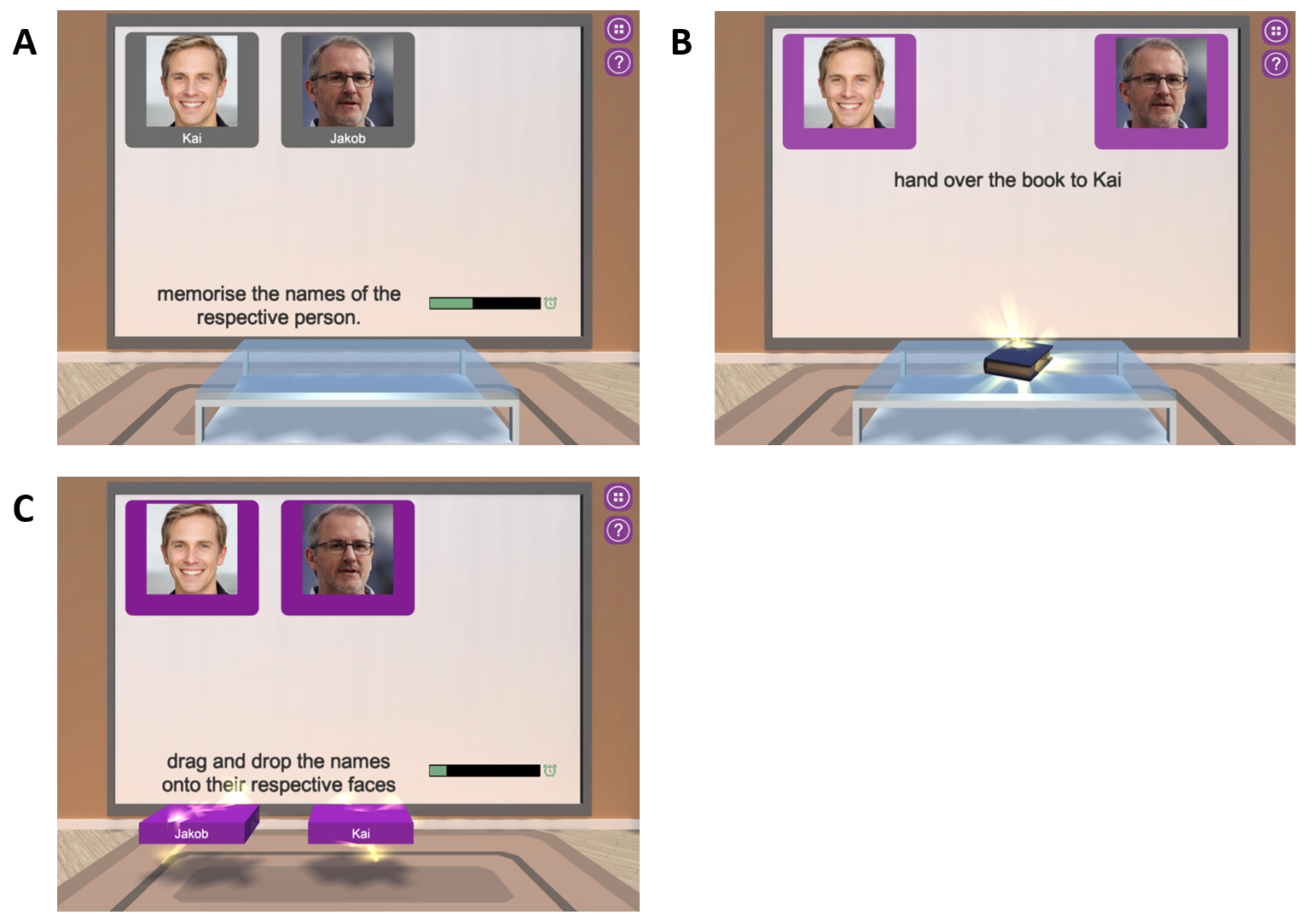


*Figure 3: What’s my name? A) Encoding phase: Multiple portraits are shown to the player. B) Recall phase 1: Items have to be handed to the correct person by dragging and dropping it to the respective portrait. C) Recall phase 2: Handing nametags to the respective persons.*

### **Shopping basket**

The goal of the “shopping basket” game is to imitate a daily life situation of running errands and to thus train functional performance in daily life. Here, an increasing number of grocery items are presented on a conveyer. First, in order to promote deeper encoding, participants are asked to rate each item as to whether they like it or not. Then, after a short delay and to probe episodic memory, the player has to recall each item freely as if writing a shopping list. Items have to be entered either via speech recognition or typed in on the keyboard. “Shopping basket” is presented in figure 4.


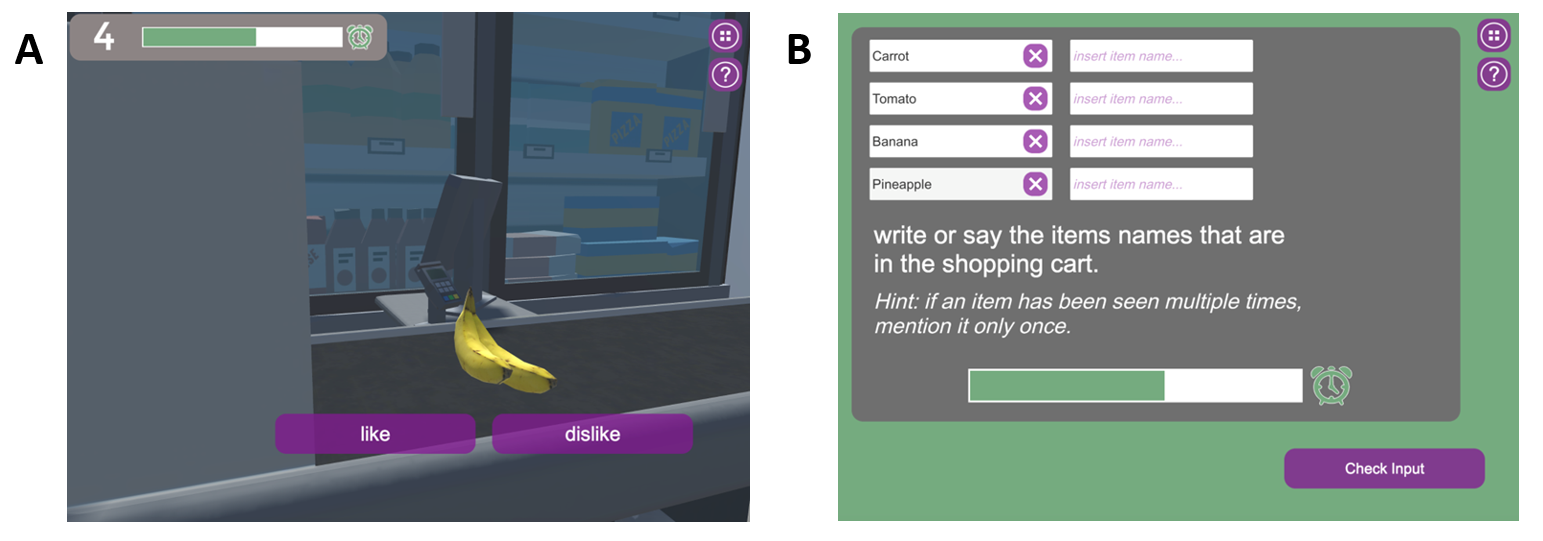


*Figure 4: Shopping basket. A) Encoding phase: Subjective rating of personal preference of different grocery items. B) Recall phase: List of previously seen items.*

**Artificial Language Learning**
We implemented a language learning game within our CCT to train the acquisition of a new language (Elvish) that is previously unknown for most players. Here, participants learn elvish words in a gamified environment. First, several words are displayed both in elbish as well as in german and a picture of the respective object is shown to ensure correct encoding. Then, each word is presented one by one and the player has to answer simple yes-and-no questions about the object (e.g. “is it human?”, “is it edible?”). Afterwards, as cued recall, the picture of each object and four possible elbish words are shown. The player has to select the correct translation. The last part of the round is a free recall condition, where the player has to freely type in the elbish translation of each word. “Artificial language learning” is presented in figure 5.


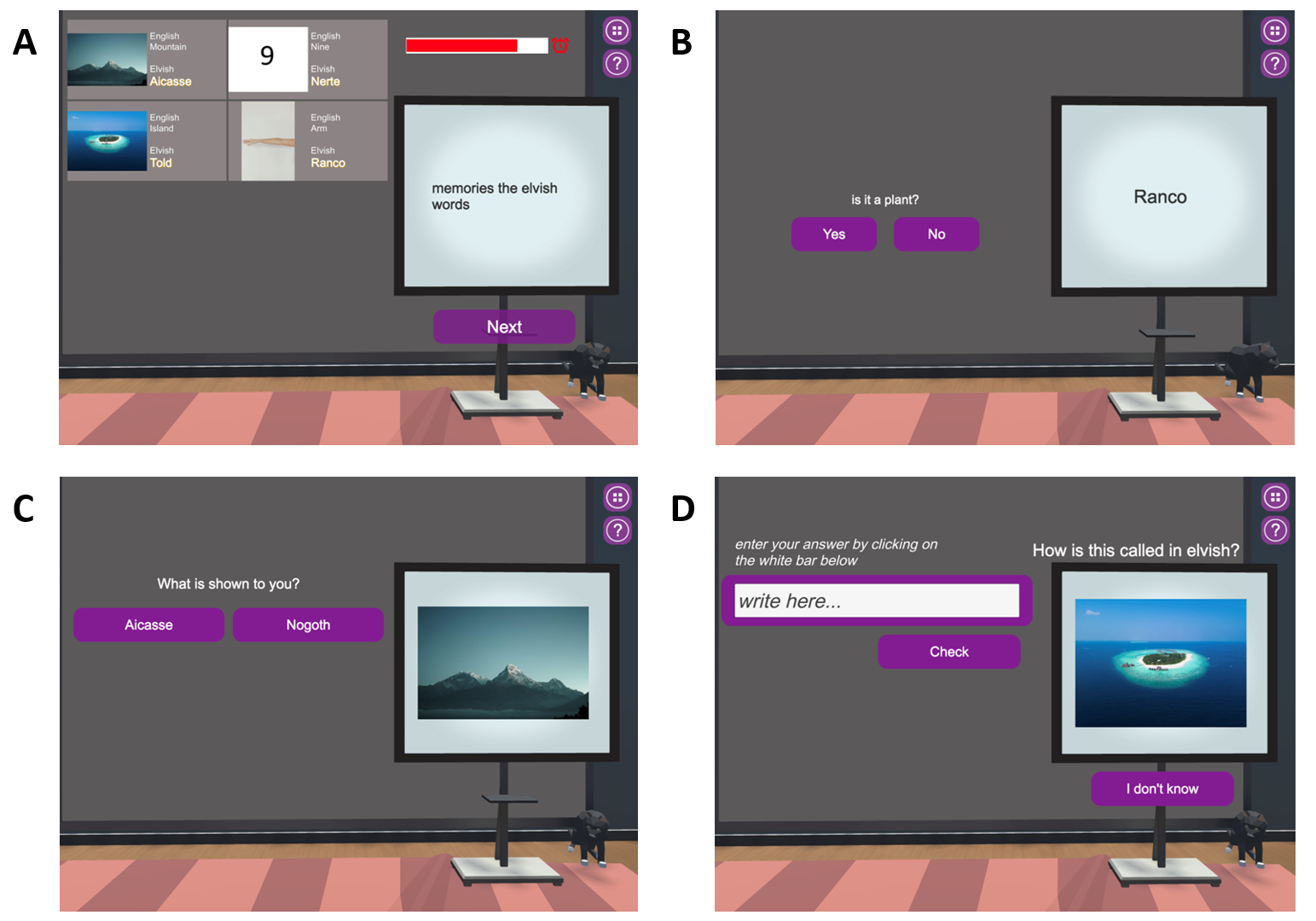


*Figure 5: Artificial language learning. A) Encoding phase: Presentation of words, both in German and in Elvish as well as a picture of the respective item. B) Cued recall phase: Simple yes or no questions about the items. C) Recognition phase: Multiple choice of possible translations. D) Free recall phase: Picture of item is presented and the Elvish translation has to be typed in.*

## **Semantic Memory**

Besides impairment of episodic memory as the hallmark of AD, the breakdown of semantic memory is well established as a consistent finding in patients, even in the early stages of the disease (1,10,11).

Semantic memory is tested and trained on a daily basis through simple tasks such as remembering names of colors, capitals of countries, or that the grass is green. More specifically, remembering names of celebrities such as politicians, scientists, sportsmen or musicians are classic examples of semantic memory. Not only are AD patients impaired on recognition, identification and naming of faces they once were familiar with (12,13) but they typically also have difficulties to correctly name objects (13,14). Early cognitive changes relevant to facial recognition and naming impairments which typically occur among patients with cortical atrophy (such as MCI or AD) can be detected through testing the ability to correctly name and/or recognize famous faces (15). Based on this early and gradually progressing (5) cognitive deficit of AD patients, training this specific memory component can have preventive aspect. Performance in semantic and lexical fluency differs in amnestic MCI and patients diagnosed with a mild stage of AD (16). More specifically, patients with AD are significantly more impaired in semantic fluency compared to phonemic fluency (17). Both fluency tasks impose comparable requirements upon executive control processes, but semantic fluency depends more on the integrity of semantic memory, thus probes the degradation of semantic storage in AD patients (17). However, there is substantial disagreement on its precise role in semantic memory considering inconsistent findings across studies.

### **Famous Faces**

“Famous Faces” trains semantic memory component by providing pictures of famous persons while challenging the player to recollect or recognize their names respectively. Portraits of famous people were used to develop the Famous Face training. The portraits selected based on the person’s popularity in the media and press at the time when the person was best-known. The game is composed of three encoding and recall conditions, depending on difficulty level. In the initial phase, the player sees pictures of famous people and has to recall their names from semantic memory by choosing from four alternatives. Secondly, the player is provided with one name and has to select the correct picture out of four possibilities. The third phase is freely recalling the famous people’s names without any cue and typing in the names solely from memory. “Famous Faces” is presented in figure 6.


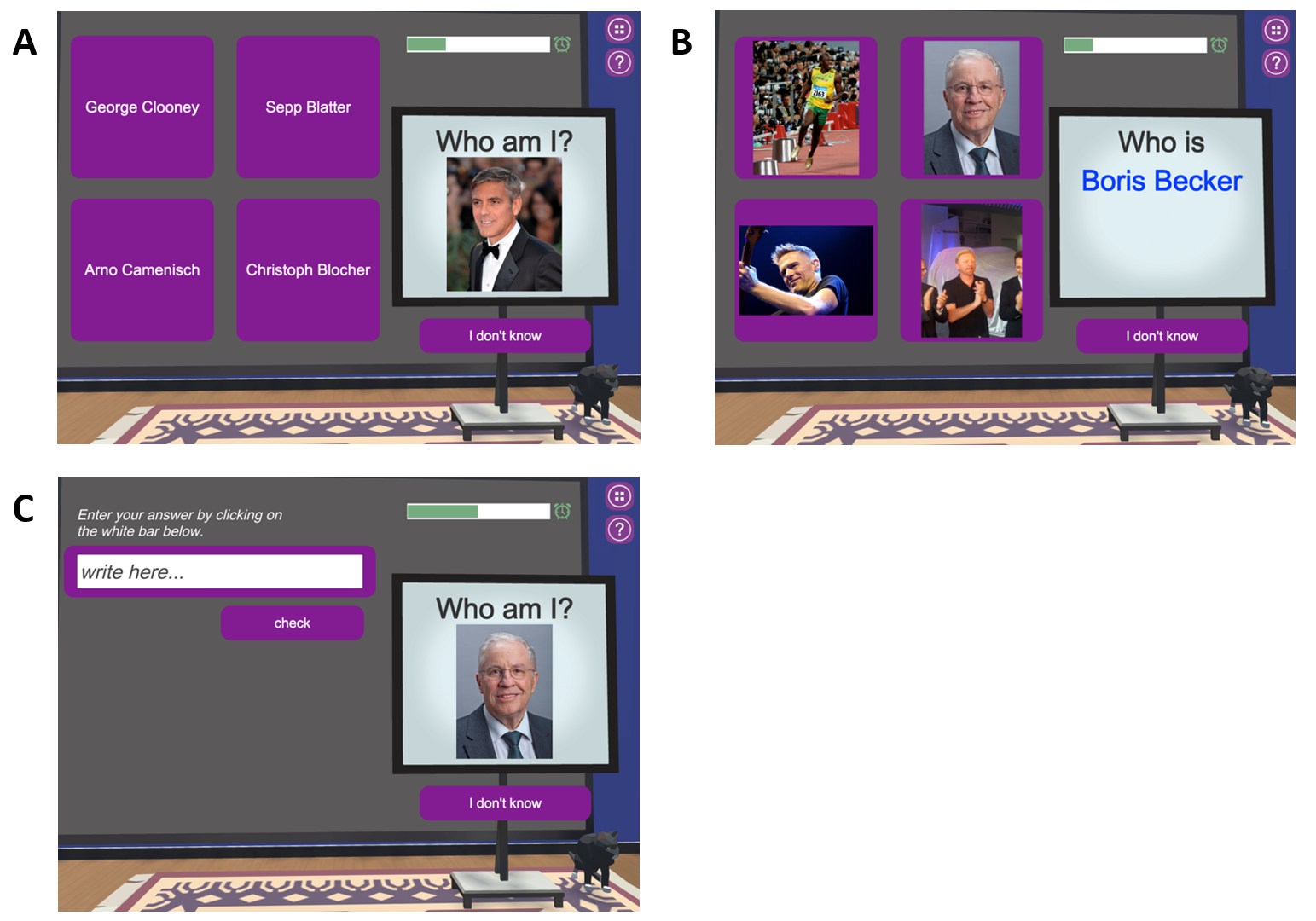


*Figure 6: Famous Faces. A) Recall phase 1: Presentation of one portrait and multiple choice selection of names. B) Recall phase 2: Presentation of one name and multiple choice selection of portraits. C) Free recall phase: Name matching the shown portraits need to be entered.*

### **Word Grid**

“Word Grid” specifically trains object naming and thus semantic memory while combing it with visuospatial exploration. “Word Grid” is based on the concept of the Graded Naming Test (GNT) (18) in which the naming of objects is tested. In “Word Grid”, participants are presented a picture of an object and asked to name it and find the respective word in the word grid. In order to find the word, participants have to challenge their semantic memory for different terms that describe the presented object. If they cannot find the word they initially thought of, they need to search for a different name for the object. The game thus trains both semantic memory as well as visuospatial abilities. “Word Grid” is presented in figure 7.


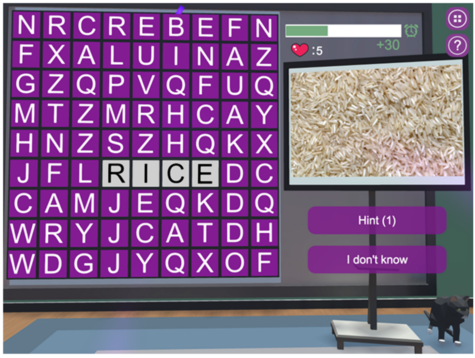


*Figure 7: Word Grid. A picture of an item is depicted on the right side of the screen and has to be found in the word grid on the left side.*

### **Pun Game**

The ability to form and retrieve links between different chunks of information is negatively affected by pathological aging (19,20). Therefore, not only the ability to name objects but also to correctly match objects to words needs to be trained in the semantic category of a CCT. The “Pun Game” is a word-picture matching task. Pictures and words are presented simultaneously and need to be matched in order to form a German double-word (e.g., “vegetable” and “broth” can be combined to “vegetable broth”). “Pun Game” is presented in figure 8.


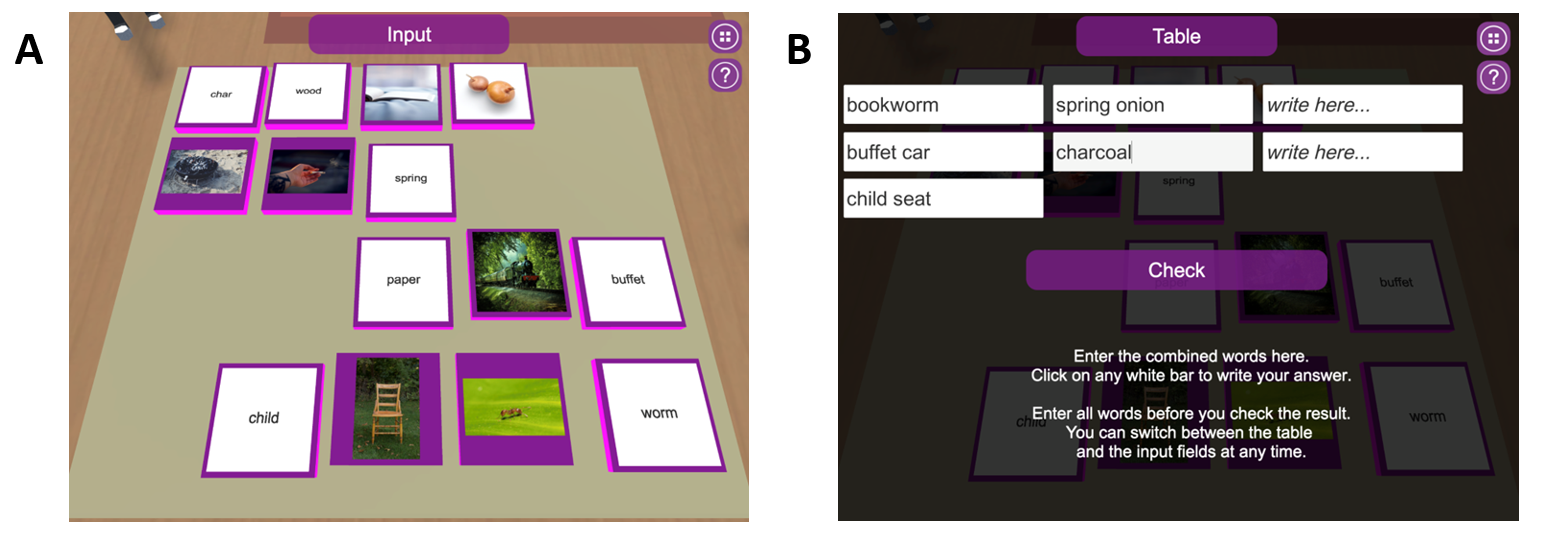


*Figure 8: Pun Game. A) Encoding phase: Cards are presented on table. Half of the items are words, half are pictures. One word and one picture item are to be combined to form a word. B) Input phase: List of paired items.*

### **Wheel of fortune**

“Wheel of fortune” is a fill the blank game based on the paradigm of the homonymous TV-show. The player first selects a category (such as “family” or “animals”) and is then presented with an empty word or phrase grid. Suitable letters have to be subsequently selected to complete the word or phrase. If a correct letter is picked, the player has another turn. If a letter is chosen incorrectly, the opponent (computer; skill based on difficulty level) can chose a letter. Initially, one letter per round is given as hint to avoid random guessing which would take up time without training any of the intended domains. “Wheel of fortune” is presented in figure 9.


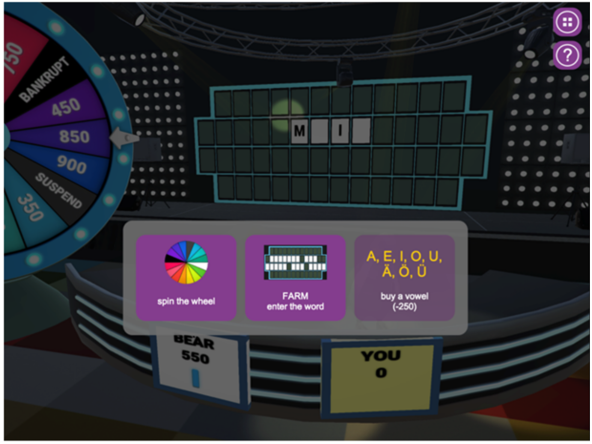


*Figure 9: Wheel of fortune. Main screen showing the wheel, the opponent and the players spirit animal (from left to right). In the background the searched word is presented in a mostly blank word grid.*

## **Visuospatial Abilities**

Visuospatial abilities entail the identification of stimuli, their location and orientation. Impairments in visuospatial abilities are a key symptom of AD, occur independently of visual impairments and can be observed in a significant portion of mild AD cases (21).

In a recent study, the specific gains and its duration of rotation training in healthy elderly was examined and a trend towards improvement was found (22). Orientation and mental rotation is a key function of visuospatial abilities. Impaired orientation in new as well as familiar environments is a frequent symptom in healthy aging, but also in AD and its prodromal stage MCI (23,24). Patients with amnestic MCI show significant difficulties in navigating compared to healthy elderly (25). Spatial disorientation is of critical relevance as it severely impairs daily life.

### **Mental Map**

The setup of “Mental Map” consists of a 3x3 grid containing one or multiple animals (depending on difficulty level) and a ball. In the encoding phase, the player is required to memorize the position of each item. Then, the animals disappear and the ball moves across the grid or the whole grid rotates around one of three axes. At the first level, there is one rotation and the number of rotations increases according to increasing levels of difficulty. Importantly, the invisible animals move analogous to the ball. Their position and orientation on the grid must be mentally tracked and then recalled by choosing the appropriate picture from a selection and dragging it to the respective square. “Mental Map” is presented in figure 10.


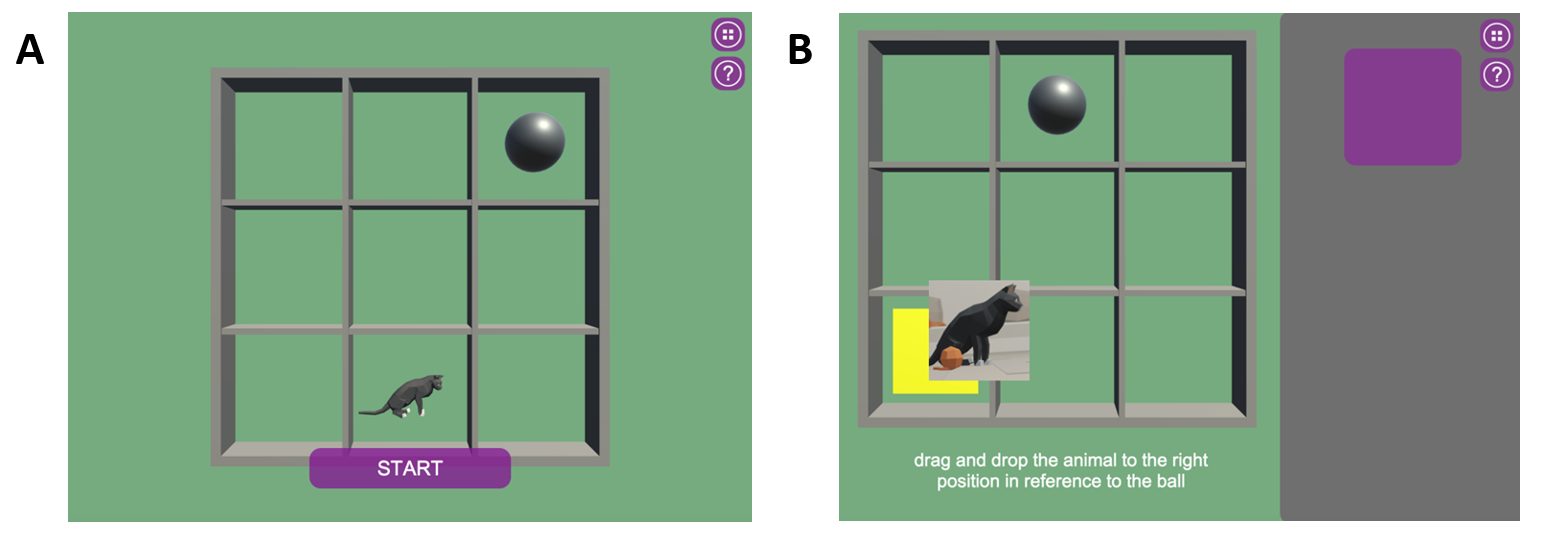


*Figure 10: Mental Map. A) Encoding phase: The grid is presented with one or more animals. B) Input phase: After either round the grid has turned and/or the ball moved around, the animal has to be placed in the respective field.*

### **Magic Camera**

The game “Magic Camera” is designed to train visuospatial perspective-taking abilities when presented with simple scenes (26). The magic camera moves around a table with randomly generated sets of items and takes photos from different angles. Participants are then presented with different pictures, of which only one depicts the previously seen scene. The wrong pictures contain small changes in the orientation of the respective items. The player can compare the initial setup on the table with the options. Answer possibilities (pictures) can be enlarged in order to have a more detailed view. “Magic Camera” is presented in figure 11.


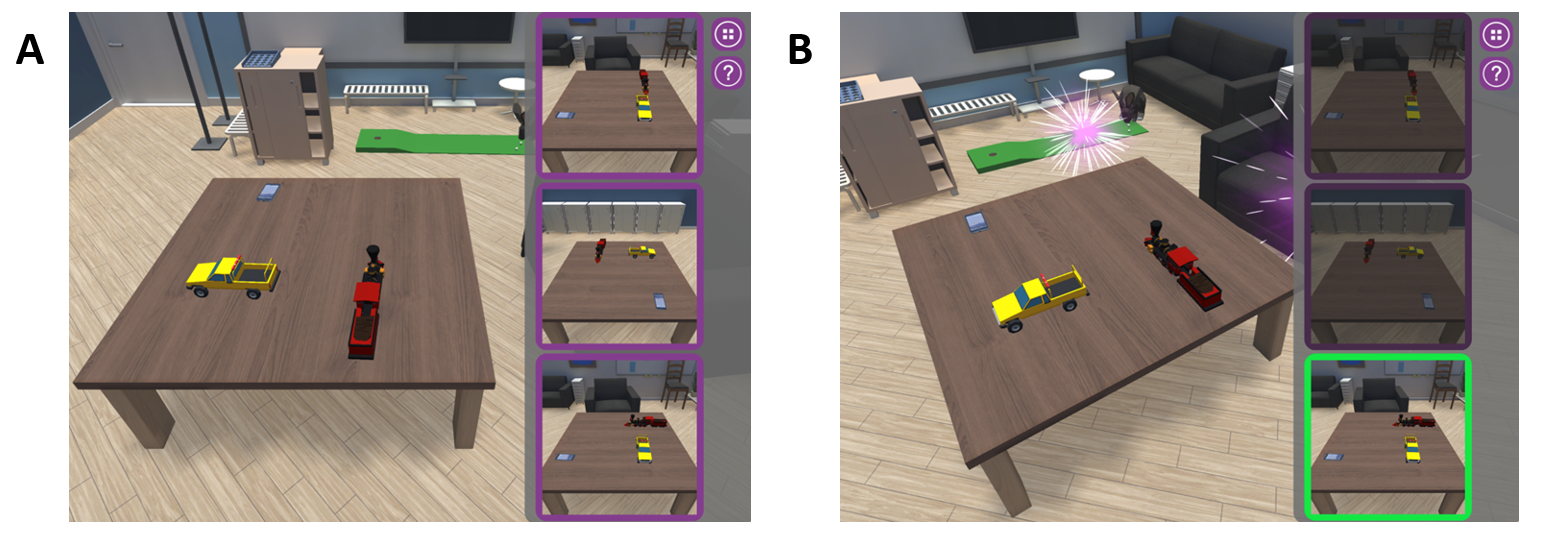


*Figure 11: Magic Camera. A) Encoding phase: The target scenery (table with items) is depicted on the left side of the screen. B) Recognition phase: The multiple answer choices are presented on the right side of the screen. The correct picture is to be selected.*

### **Wine Cellar**

“Wine Cellar” trains visuospatial navigation ability inspired by Götz and colleagues (27). First, the player is passively navigated through a wine cellar and must memorize the path and its respective turns leading to the player’s spirit animal as goal. Then, the player is taken back to the start and has to actively find the way to the spirit animal by indicating the correct left and right turns. “Wine Cellar” is presented in figure 12.


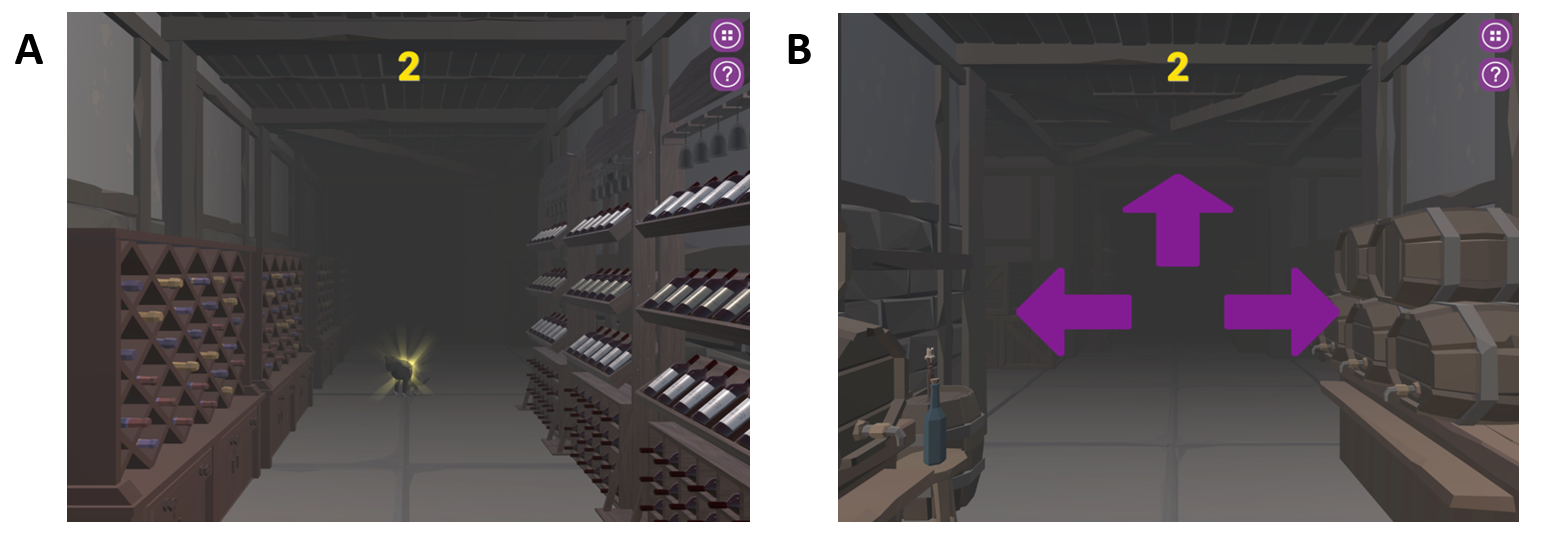


*Figure 12. Wine Cellar. A) Encoding phase: Passive walk-through the wine cellar towards the spirit animal. B) Recall phase: Selectable arrows to replicate the previously seen path.*

### **Mansion Game**

One’s ability to form mental representations of the surroundings is of critical importance in spatial navigation and orientation. A novel computerized training program to improve spatial orientation was designed by Binder (28) and colleagues, showing the feasibility of training visuospatial orientation skills in healthy volunteers. Therefore, we developed the “Mansion Game”. Initially, in order to build a “cognitive map” of the scene. The players are given the opportunity to familiarize themselves with the surroundings by walking through the mansion. Hereby, as a preliminary goal, the player travels back and forth between environmental landmarks with the purpose of finding color-coded keys and a chest which can be opened with the keys. In a second stage to probe spatial abilities as well as episodic memory, the player has to remember where the keys were to be found. He/she has to transform its 3D understanding of the mansion into a 2D map and indicate the locations of the previously collected keys in the map. “Mansion Game” is presented in figure 13.


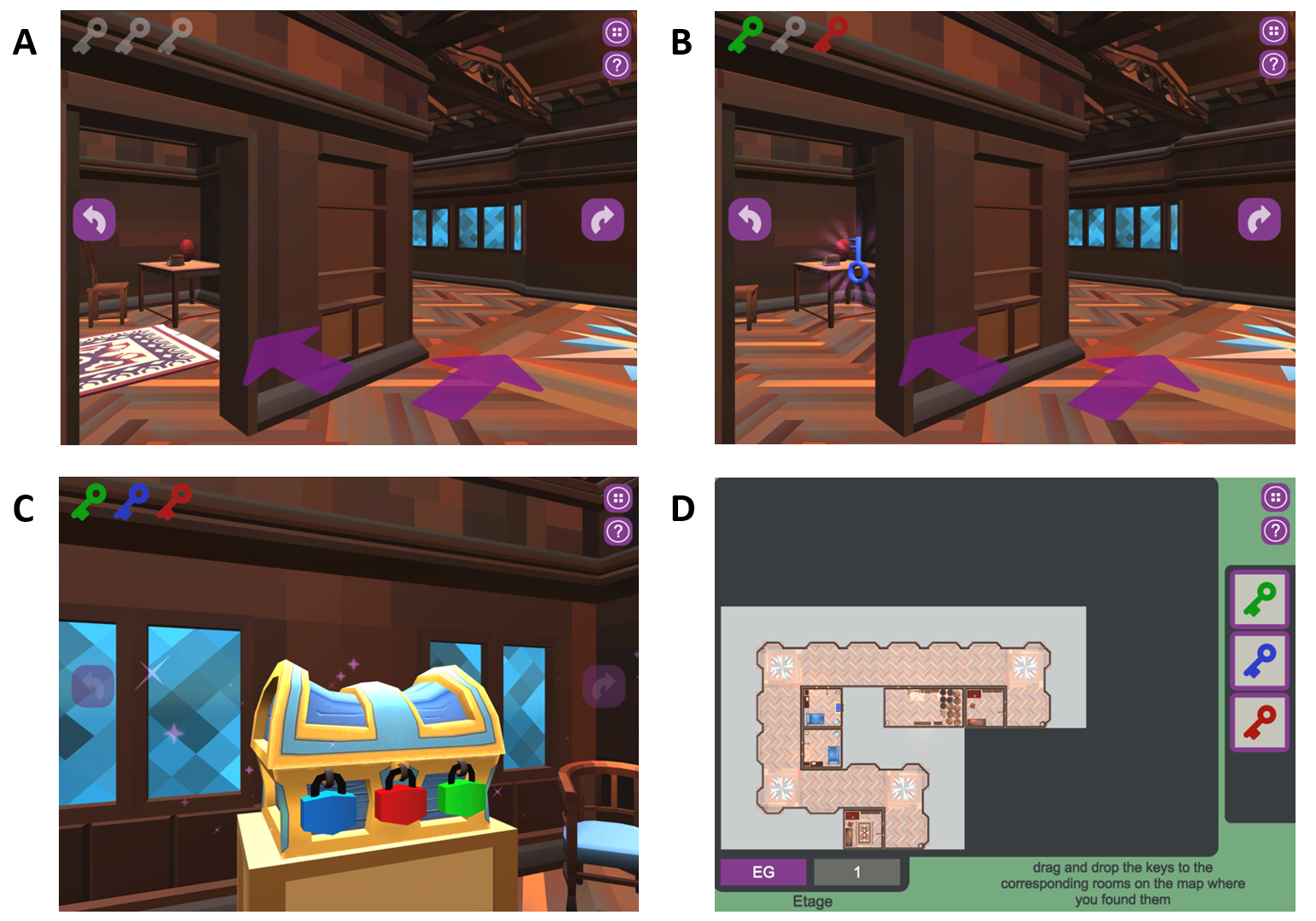


*Figure 13. Mansion Game. A) Active walk through the mansion. Directions can be chosen by tapping on the respective arrows. B) Finding one of the keys in a room. C) Finding the to-be-opened chest. A)-C) constitute the 3D - encoding phase. D) Recall phase: 2D map of the mansion. The keys have to be dragged and dropped into the rooms they were previously found in.*

## **Working Memory**

Several studies have described working memory as crucial element in cognitive training since it is considered a key component of higher cognitive functions (29,30). Working memory allows the maintenance and manipulation of online information and supports multiple complex cognitive tasks including language comprehension, mathematical abilities, and reasoning (31). Aging and neurodegenerative disease can negatively affect working memory. Several studies found positive effects on cognition of working memory training in different age ranges and patient groups (32,33).

### **Memory Bears**

Both MCI and AD patients show a lower performance in classic tests assessing verbal and visuospatial working memory such as digit and spatial span tests backwards, with spatial span backwards being sensitive to severity of cognitive impairment (34,35). We developed “Memory Bears” by transforming the spatial span test into a gamified training for visual-spatial working memory. Here, bears in a forest light up in a specific sequence and player is required to tap the bears in reversed order. “Memory Bears” is presented in figure 14.


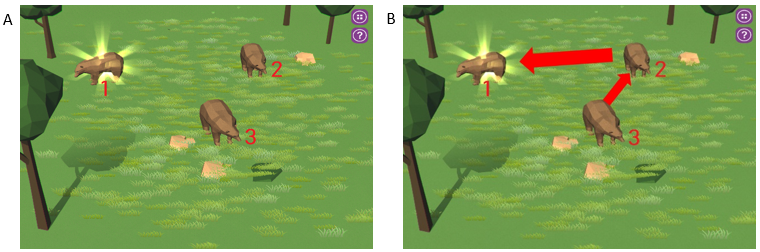


*Figure 14: Memory Bears. A) Encoding phase. Bears glow in a certain sequence. B) Recall phase. The sequence of bears has to be replicated in reverse order.*

##### **Safari**

“Safari” is based on the paradigm of the well-studied n-back task, which has been proven to be a useful tool in cognitive training across different age groups (36,37). Here, working memory is continuously engaged, first at a low, then increasingly higher workload (38). This may then subsequently be transferred into more general tasks that rely on the integrity of working memory skills (39). Subjects are presented with a sequence of animals through a virtual camera lens and take a picture every time an animal matches another animal displayed *n* trials ago. Initially the game starts with one-back trials and difficulty increases to n+1 throughout correctly completing several rounds. “Safari” is presented in figure 15.


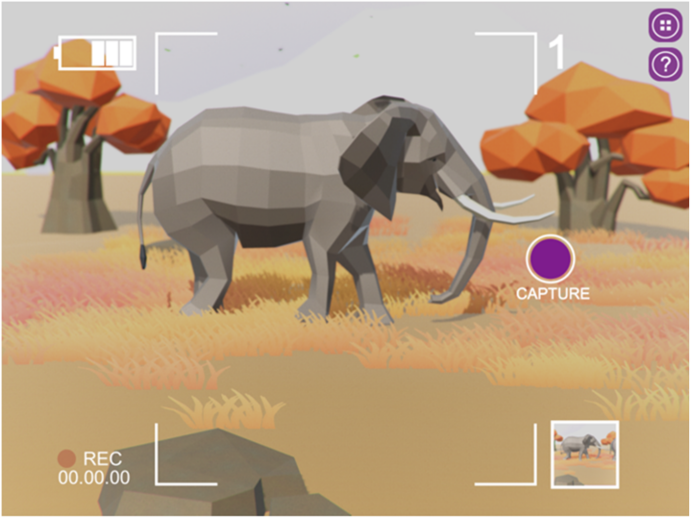


*Figure 15: Safari. To be remembered animal (i.e. giraffe) walking from left to right through the landscape.*

### **Quiz Game**

Both mental flexibility and attention are crucial to a well-functioning memory and research supports its role as a valuable training tool to enhance working memory (9). We developed the “Quiz Game” to train those working memory components according to Robert et al. (9). First, participants see a sequence of rather simple questions (e.g. “What is the capital of France?”, “Which month comes after January?”). Secondly, the respective answers need to be remembered in the correct order. Subsequently, the order needs to be replicated by selecting from a multiple choice set of answers. “Quiz Game” is presented in figure 16.


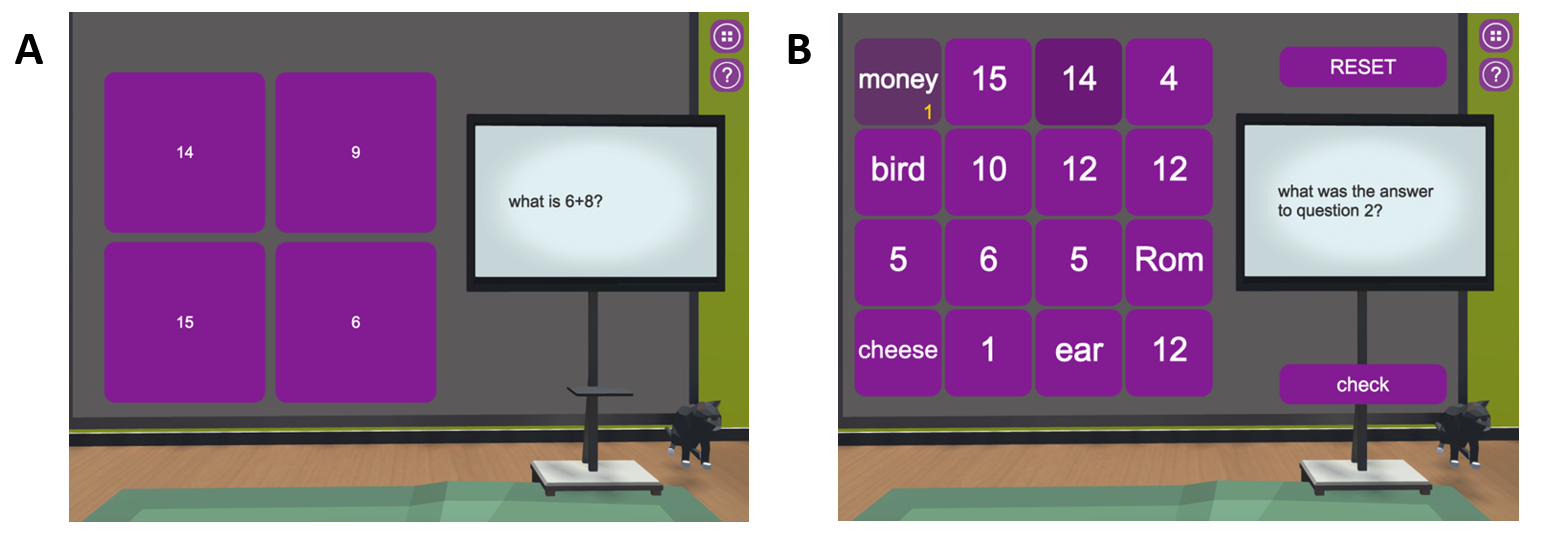


*Figure 16. Quiz Game. A) Question phase: Simple questions with multiple choice answers. B) Recall phase: Replication of the sequence of answers.*

### **Animal Park**

“Animal Park” trains speed of processing and memory span, while additionally containing a spatial and temporal working memory component. First, participants must indicate whether pictures of animals are presented upright or upside down and simultaneously memorize the sequence and spatial position in which they appeared. Afterwards, the animals need to be dragged to their initial position in the previously seen order. “Animal Park” is inspired by Studer-Lüthi et al. (40) and is presented in figure 17.


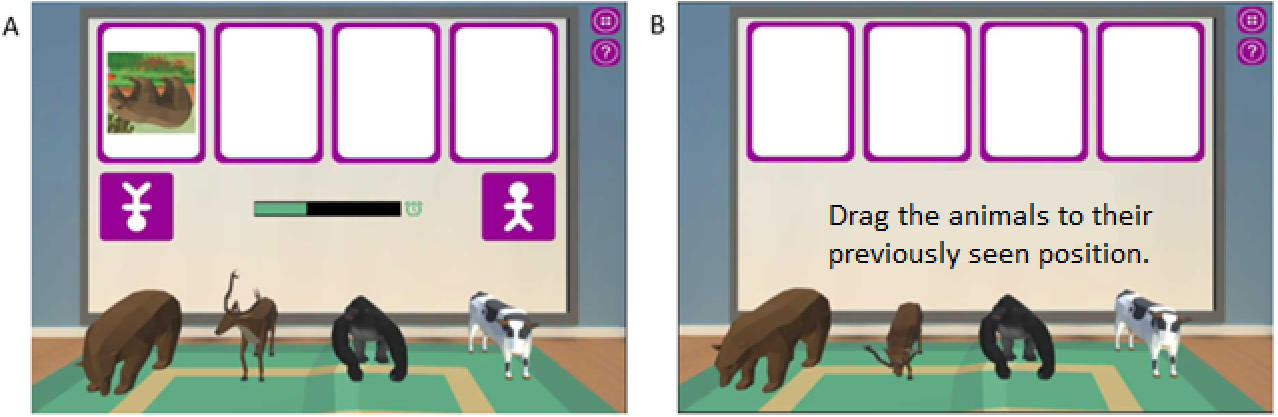


*Figure 17: Animal Park. A) Encoding phase: Animals are presented either upright or upside down in the upper part of the screen. Their orientation is to be categorized. B) Recall phase: Animals are to be placed in the previously seen order into the respective fields.*

# **References:**

1. Dudas RB, Clague F, Thompson SA, Graham KS, Hodges JR. Episodic and semantic memory in mild cognitive impairment. Neuropsychologia. 2005;43(9):1266–76.

2. Rubiño J, Andrés P. The Face-Name Associative Memory test as a tool for early diagnosis of alzheimer’s disease. Front Psychol. 2018;9(AUG):1–5.

3. Tromp D, Dufour A, Lithfous S, Pebayle T, Després O. Episodic memory in normal aging and Alzheimer disease: Insights from imaging and behavioral studies. Ageing Res Rev [Internet]. 2015;24:232–62. Available from: http://dx.doi.org/10.1016/j.arr.2015.08.006

4. Degenszajn J, Caramelli P, Caixeta L, Nitrini R. Encoding process in delayed recall impairment and rate of forgetting in Alzheimer’s disease. Arq Neuropsiquiatr. 2001;59(2 A):171–4.

5. Tak SH, Hong SH. Face-name memory in Alzheimer’s disease. Geriatr Nurs (Minneap) [Internet]. 2014;35(4):290–4. Available from: http://dx.doi.org/10.1016/j.gerinurse.2014.03.004

6. Bahar-Fuchs A, Clare L, Woods B. Cognitive training and cognitive rehabilitation for mild to moderate Alzheimer’s disease and vascular dementia. Cochrane Database Syst Rev. 2013;2013(6).

7. Clare L, Wilson BA, Carter G, Roth I, Hodges JR. Relearning face-name associations in early Alzheimer’s disease. Neuropsychology. 2002;16(4):538–47.

8. Kesslak JP, Nackoul K, Sandman CA. Memory training for individuals with Alzheimer’s disease improves name recall. Behav Neurol. 1997;10(4):137–42.

9. Robert P, Manera V, Derreumaux A, Montesino MFY, Leone E, Fabre R, et al. Efficacy of a web app for cognitive training (MEMO) regarding cognitive and behavioral performance in people with neurocognitive disorders: Randomized controlled trial. J Med Internet Res. 2020;22(3):1–11.

10. Hodges JR, Patterson K. Is semantic memory consistently impaired early in the course of Alzheimer’s disease? Neuroanatomical and diagnostic implications. Neuropsychologia. 1995;33(4):441–59.

11. Chertkow H, Bub D. Semantic memory loss in dementia of alzheimer’s type: What DO various MEASURES measure? Brain. 1990;113(2):397–417.

12. Greene JDW, Hodges JR. Identification of famous faces and famous names in early Alzheimer’s disease: Relationship to anterograde episodic and general semantic memory. Brain. 1996;119(1):111–28.

13. Ahmed S, Arnold R, Thompson SA, Graham KS, Hodges JR. Naming of objects, faces and buildings in mild cognitive impairment. Cortex. 2008;44(6):746–52.

14. Williams BW, Mack W, Henderson VW. Boston Naming Test in Alzheimer’s disease. Neuropsychologia. 1989;27(8):1073–9.

15. Rahmani F, Fathi M, Kazemi M, Bahadori E. Recognition of famous and unfamiliar faces among patients suffering from amnesia mild cognitive impairment (AMCI) and Alzheimer’s disease. Iran J Psychiatry. 2019;14(3):227–35.

16. Lonie JA, Herrmann LL, Tierney KM, Donaghey C, O’Carroll R, Lee A, et al. Lexical and semantic fluency discrepancy scores in aMCI and early Alzheimer’s disease. J Neuropsychol. 2009;3(1):79–92.

17. Henry JD, Crawford JR, Phillips LH. Verbal fluency performance in dementia of the Alzheimer’s type: A meta-analysis. Neuropsychologia. 2004;42(9):1212–22.

18. McKenna P, Warrington EK. Testing for nominal dysphasia. J Neurol Neurosurg Psychiatry. 1980;43(9):781–8.

19. Klink K, Peter J, Wyss P, Klöppel S. Transcranial Electric Current Stimulation During Associative Memory Encoding: Comparing tACS and tDCS Effects in Healthy Aging. Front Aging Neurosci. 2020;12(March):1–12.

20. Old SR, Naveh-Benjamin M. Differential effects of age on item and associative measures of memory: A meta-analysis. Vol. 23, Psychology and Aging. Naveh-Benjamin, Moshe: Department of Psychological Sciences, University of Missouri, 106 McAlester Hall, Columbia, MO, US, 65211, NavehbenjaminM@missouri.edu: American Psychological Association; 2008. p. 104–18.

21. Quental NBM, Brucki SMD, Bueno OFA. Funções visoespaciais na doença de alzheimer de intensidade leve: Estudo preliminar. Dement e Neuropsychol. 2009;3(3):234–40.

22. Meneghetti C, Carbone E, Di Maggio A, Toffalini E, Borella E. Mental rotation training in older adults: The role of practice and strategy. Vol. 33, Psychology and Aging. Meneghetti, Chiara: Department of General Psychology, University of Padova, Via Venezia 8, Padova, Italy, 35131, chiara.meneghetti@unipd.it: American Psychological Association; 2018. p. 814–31.

23. Flicker C, Ferris SH, Crook T, Reisberg B, Bartus RT. Equivalent spatial-rotation deficits in normal aging and Alzheimer’s disease. J Clin Exp Neuropsychol. 1988 Aug;10(4):387–99.

24. Quimas Molina da Costa R, Pompeu JE, Pereira de Viveiro LA, Brucki SMD. Spatial orientation tasks show moderate to high accuracy for the diagnosis of mild cognitive impairment: A systematic literature review. Arq Neuropsiquiatr. 2020;78(11):713–23.

25. Peter J, Sandkamp R, Minkova L, Schumacher L V., Kaller CP, Abdulkadir A, et al. Real-world navigation in amnestic mild cognitive impairment: The relation to visuospatial memory and volume of hippocampal subregions. Neuropsychologia [Internet]. 2018;109(September 2017):86–94. Available from: https://doi.org/10.1016/j.neuropsychologia.2017.12.014

26. McLaren-Gradinaru M, Burles F, Dhillon I, Retsinas A, Umiltà A, Hannah J, et al. A Novel Training Program to Improve Human Spatial Orientation : Preliminary Findings. 2020;14(January):1–11.

27. Götz, U., Kocher, M., Bauer, R., Müller, C., Meilick B. Challenges for Serious Game Design. Designing the Game-Based Neurocognitive Research Software “Hotel Plastisse.” Games and Learning Alliance. Springer.; 2016. 323–328 p.

28. Binder JC, Zöllig J, Eschen A, Mérillat S, Röcke C, Schoch SF, et al. Multi-domain training in healthy old age: Hotel Plastisse as an iPad-based serious game to systematically compare multi-domain and single-domain training. Front Aging Neurosci. 2015;7(JUL).

29. Boujut A, Mellah S, Lussier M, Maltezos S, Verty LV, Bherer L, et al. Assessing the effect of training on the cognition and brain of older adults: Protocol for a three-arm randomized double-blind controlled trial (ACTOP). JMIR Res Protoc. 2020;9(11).

30. Engle RW. Working Memory and Executive Attention: A Revisit. Perspect Psychol Sci. 2018;13(2):190–3.

31. Baddeley AD, Hitch G. The social design of virtual worlds: constructing the user and community through code. Internet Res Annu Sel Pap from Assoc Internet Res Conf 2000-2002, Vol 1. 1974;260–8.

32. Vivas AB, Ypsilanti A, Ladas AI, Kounti F, Tsolaki M, Estévez AF. Enhancement of Visuospatial Working Memory by the Differential Outcomes Procedure in Mild Cognitive Impairment and Alzheimer’s Disease. Front Aging Neurosci. 2018;10(November):1–7.

33. Hill NTM, Mowszowski L, Naismith SL, Chadwick VL, Valenzuela M, Lampit A. Computerized cognitive training in older adults with mild cognitive impairment or dementia: A systematic review and meta-analysis. Am J Psychiatry. 2017;174(4):329–40.

34. Kessels RPC, Overbeek A, Bouman Z. Avaliação da memória de trabalho verbal e visuoespacial no comprometimento cognitivo leve e na doença de Alzheimer. Dement e Neuropsychol. 2015;9(3):301–5.

35. Wiechmann A, Hall JR, O’Bryant SE. The utility of the spatial span in a clinical geriatric population. Neuropsychol Dev Cogn Sect B, Aging, Neuropsychol Cogn. 2011 Jan;18(1):56–63.

36. Blocker KA, Wright TJ, Boot WR. Gaming preferences of aging generations. Gerontechnology. 2014;12(3):174–84.

37. Lilienthal L, Tamez E, Shelton JT, Myerson J, Hale S. Dual n-back training increases the capacity of the focus of attention. Psychon Bull Rev. 2013;20(1):135–41.

38. Coulacoglou C, Saklofske DH. Executive Function, Theory of Mind, and Adaptive Behavior. Psychometrics and Psychological Assessment. 2017. 91–130 p.

39. Jaeggi SM, Buschkuehl M, Jonides J, Perrig WJ. Improving fluid intelligence with training on working memory. Proc Natl Acad Sci U S A. 2008;105(19):6829–33.

40. Studer-Lüthi B, Meier B, Frey T, Kodzhabashev S. iHirn. A collection of online cognitive training tasks. Switz Univ Bern.götz. 2017;
