# Supplementary material for: Can a serious game-based cognitive training attenuate cognitive decline related to Alzheimer’s disease? Protocol for a randomized controlled trial
Source: BMC Psychiatry. 2022 Aug 12;22:552. doi: 10.1186/s12888-022-04131-7 (PMC9373273; doi:10.1186/s12888-022-04131-7)
Supplement: Supplementary file 2 — Additional file 2. Functional MRI tasks. [file 12888_2022_4131_MOESM2_ESM.docx]

# **Additional file 2. Functional MRI tasks**

During the episodic memory (EM) functional magnetic resonance imaging (fMRI) task, six blocks with five face-occupation pairs are presented. In a first block the participants indicate, whether the face and the occupation match. This rating is entirely subjective and choices cannot be correct or false. The second block consists of a control condition where participants will rate five white head shapes per block as either male or female. Then cued recall is tested by the presentation of the faces and the question whether the occupation requires a university degree or an apprenticeship. The final block consists of a recognition test were the faces are presented together with two occupations and the participant has to choose the correct one (i.e., the occupation which was presented during the encoding phase). The EM task is depicted in figure 1. The design is adapted from a task used in a previous study (1).


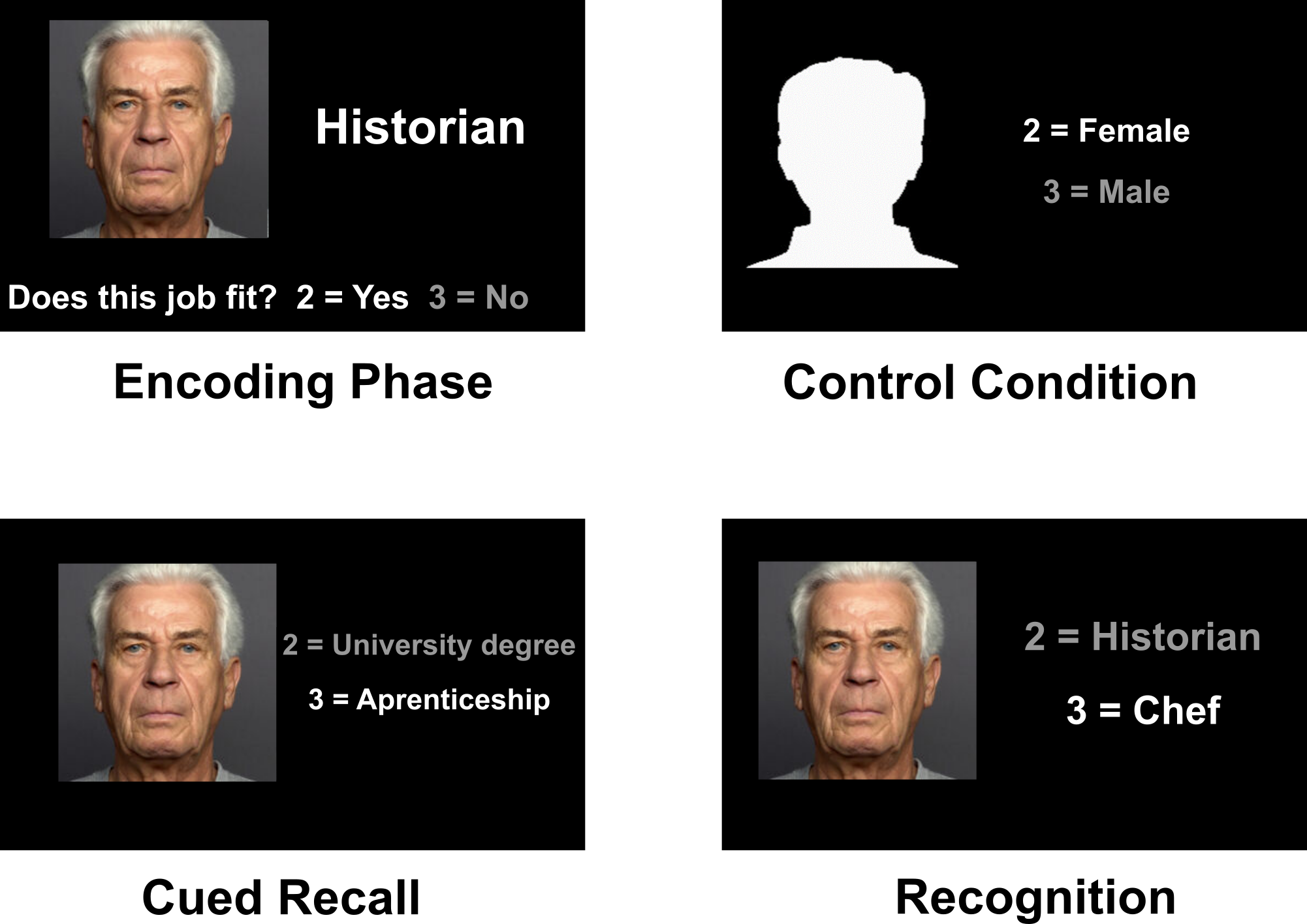


Figure 1 Face-occupation task. A face is shown together with an occupation (decision: the occupation fits the person yes or no), followed by a control condition (decision if shape is male or female) and a cued recall (decision if a university degree or an apprenticeship is necessary for the occupation of this person). Finally, participants have to choose the correct occupation between two possible answers (recognition). Each block consists of five face-occupation pairs, 15 in total, which are shown twice across 30 blocks in six runs. The faces used in this task are part of the FACES database (2). The use of the displayed face is permitted (2) for illustrating research methodology.

Each face-occupation pair is shown twice with a break of two runs before the second presentation. The stimuli will be randomly assigned to a run and be presented for 3.75 seconds each. After button press the given answer will be indicated with grey color and the stimuli remains on screen until the 3.75 seconds are over to ensure comparable encoding and recall conditions for all participants. The inter-stimulus interval is set to 0.5 seconds. In total 15 face-occupation pairs will be presented during the EM task in six runs. The faces used in this task are part of the FACES database (2).

For the EM task, four parallel versions with different sets of faces and occupations for each assessment will be used.

In the spatial ability (SA) task four conditions exist: translation, rotation and corresponding control conditions. In the translation and rotation condition, participants will see two puzzle pieces made of polygon shapes and have to indicate if they fit together to form a square. In the translation condition this can be achieved by mentally sliding the pieces to each other while in the rotation condition the participant have to mentally rotate one piece for 90 degrees to decide if they fit together. In the control condition participants see two black rectangles in the size of the puzzle pieces with one little grey square on each piece. Corresponding to the translation and rotation condition, the two rectangles will either be presented in the same orientation (luminance translation condition) or with one rectangle rotated (luminance rotation condition). The participants have to indicate whether the grey squares have exactly the same color. The design is adapted from a fMRI task used in a previous study (3). The next trial appears when an answer is indicated or after 2.5 seconds without an answer. Therefore, the amount of stimuli varies between participants and blocks. The duration of each block will be set to 24 seconds with a total of eight blocks in two runs. The inter-stimulus interval is set to 0.5 seconds. The SA task is depicted in figure 2.


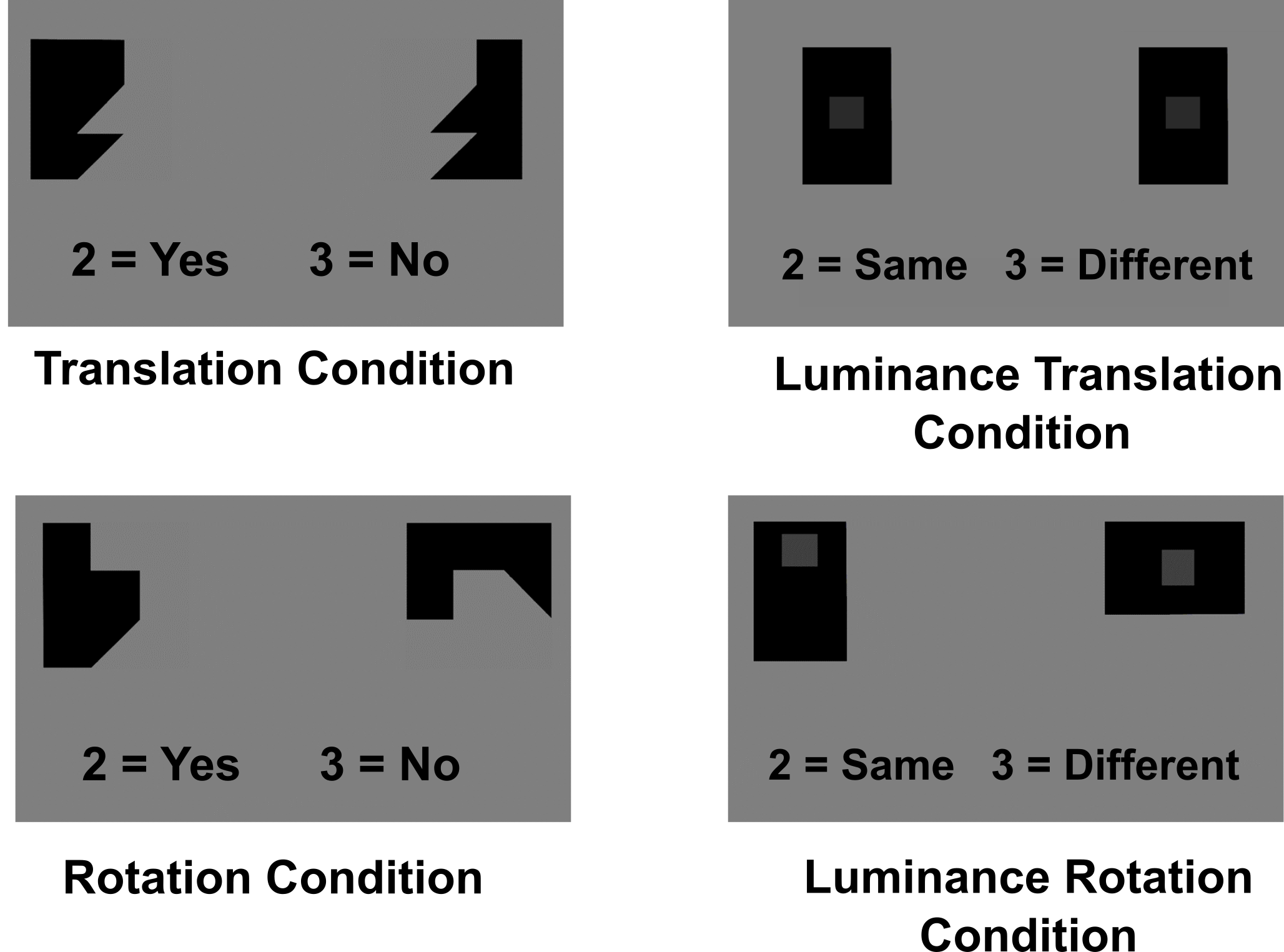


Figure 2: Spatial Ability Task. The task consists of four blocks. In the translation and rotation condition block participants have to either translate or rotate puzzle pictures mentally to decide if the pieces would fit together to build a rectangle. In control condition blocks, participants indicate if the two grey squares in the black rectangles have the same colour. Each block takes 24 seconds; the number of presented stimuli varies as the task is self-paced. The blocks are presented twice, i.e., eight blocks are shown in total in two runs. Copyright permission to use this picture was obtained.

The SA task starts with a translation block, while the parallel version will start with a luminance block. This parallel version will be used in the second and fourth assessment to avoid habituation effects through repeated testing in the same block sequence.

During the resting state fMRI, a white fixation cross on a black background will be presented.

Resting state fMRI will be performed with eyes open and fixation (EOF) which increases the strength of network connectivity compared to eyes closed and reduces the amount of eye movements (4). Additionally, EOF in resting state fMRI increases reliability (5) and wakefulness (6). Also arterial spin labelling will be performed without concurrent task and eyes open.

# **References**

1. Klink K, Peter J, Wyss P, Klöppel S. Transcranial Electric Current Stimulation During Associative Memory Encoding: Comparing tACS and tDCS Effects in Healthy Aging. Front Aging Neurosci. 2020;12(March):1–12.

2. Ebner NC, Riediger M, Lindenberger U. FACES-a database of facial expressions in young, middle-aged, and older women and men: Development and validation. Behav Res Methods. 2010;42(1):351–62.

3. Seydell-Greenwald A, Ferrara K, Chambers CE, Newport EL, Landau B. Bilateral parietal activations for complex visual-spatial functions: Evidence from a visual-spatial construction task. Neuropsychologia [Internet]. 2017;106(September):194–206. Available from: http://dx.doi.org/10.1016/j.neuropsychologia.2017.10.005

4. Van Dijk KRA, Hedden T, Venkataraman A, Evans KC, Lazar SW, Buckner RL. Intrinsic functional connectivity as a tool for human connectomics: Theory, properties, and optimization. J Neurophysiol. 2010;103(1):297–321.

5. Patriat R, Molloy EK, Meier TB, Kirk GR, Nair VA, Meyerand ME, et al. The effect of resting condition on resting-state fMRI reliability and consistency: A comparison between resting with eyes open, closed, and fixated. Neuroimage [Internet]. 2013;78:463–73. Available from: http://dx.doi.org/10.1016/j.neuroimage.2013.04.013

6. Zou Q, Miao X, Liu D, Wang DJJ, Zhuo Y, Gao JH. Reliability comparison of spontaneous brain activities between BOLD and CBF contrasts in eyes-open and eyes-closed resting states. Neuroimage. 2015;121:91–105.
