# Supplementary material for: Can a serious game-based cognitive training attenuate cognitive decline related to Alzheimer’s disease? Protocol for a randomized controlled trial
Source: BMC Psychiatry. 2022 Aug 12;22:552. doi: 10.1186/s12888-022-04131-7 (PMC9373273; doi:10.1186/s12888-022-04131-7)
Supplement: Supplementary file 3 — Additional file 3. Expectations towards cognitive training questionnaire. [file 12888_2022_4131_MOESM3_ESM.docx]

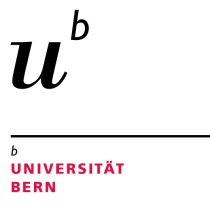
UNIVERSITÄTSKLINIK FÜR ALTERSPSYCHIATRIE UND PSYCHOTHERAPIE

| Cognitive Training Study |
| --- |

^

ID. Investigator:

Date / / (TT/MM/JJJJ) Time: :

Expectations towards cognitive training

With the following questions, we want to evaluate your current expectations towards the cognitive training.
Please mark the most suitable answer with a cross.

How much do you expect your memory performance to improve with 3 months of training?

| 1 | 2 | 3 | 4 | 5 | 6 | 7 | 8 | 9 |
| --- | --- | --- | --- | --- | --- | --- | --- | --- |

Not at all a little bit strongly

How convinced would you be right now if you were recommending the cognitive training to a firend?

| 1 | 2 | 3 | 4 | 5 | 6 | 7 | 8 | 9 |
| --- | --- | --- | --- | --- | --- | --- | --- | --- |

Not al tll a little bit strongly

What do you expect the improvement in memory performance to be after three months of training?

| 10% | 20% | 30% | 40% | 50% | 60% | 70% | 80% | 90% | 100% |
| --- | --- | --- | --- | --- | --- | --- | --- | --- | --- |
