# Supplementary material for: Can a serious game-based cognitive training attenuate cognitive decline related to Alzheimer’s disease? Protocol for a randomized controlled trial
Source: BMC Psychiatry. 2022 Aug 12;22:552. doi: 10.1186/s12888-022-04131-7 (PMC9373273; doi:10.1186/s12888-022-04131-7)
Supplement: Supplementary file 4 — Additional file 4. Subjective cognitive performance questionnaire (Self-assessment). [file 12888_2022_4131_MOESM4_ESM.docx]

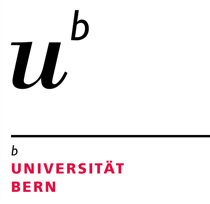
UNIVERSITÄTSKLINIK FÜR ALTERSPSYCHIATRIE UND PSYCHOTHERAPIE

| Cognitive Training Study |
| --- |

ID: Investigator:

Date / / (TT/MM/JJJJ) Time: :

Subjective cognitive performance (Self-assessment)

Do you feel that your cognitive performance has changed in the **last 12 months**? Please rate how much each area has been affected. If you have not noticed any change, please tick "0" in each case.

Memory in general

„much worse“ „much better“

-5 -4 -3 -2 -1 0 1 2 3 4 5

Remembering things about family members and friends (e.g., birthdays, addresses, occupations).

„much worse“ „much better“

-5 -4 -3 -2 -1 0 1 2 3 4 5

Remembering events that happened a long time ago

„much worse“ „much better“

-5 -4 -3 -2 -1 0 1 2 3 4 5

Remembering events that happened recently

„much worse“ „much better“

-5 -4 -3 -2 -1 0 1 2 3 4 5

Learning new things in general

„much worse“ „much better“

-5 -4 -3 -2 -1 0 1 2 3 4 5

Attention ("the ability to focus on one thing")

„much worse“ „much better“

-5 -4 -3 -2 -1 0 1 2 3 4 5

If you have noticed changes in any of the above areas, are you concerned about them?

„no, not at all“ „yes, very much“

0 1 2 3 4 5 6 7 8 9 10
